# Genome-wide identification and expression profiling of the histone deacetylase gene family in *Fusarium oxysporum*

**DOI:** 10.3897/imafungus.17.168980

**Published:** 2026-01-29

**Authors:** Hong-Xin Liao, Jin-Rui Wen, Hong-Mei Shi, Huan-Qi Cun, Yun-Ju Hong, Zhang-Feng Hu, Fu-Rong Xu, Sulukkana Noiprasert, Kanyaphat Apiwongsrichai, Xiao-Yun Liu, Xian Dong

**Affiliations:** 1 Yunnan Key Laboratory of Chinese Medicine Processing, Yunnan University of Chinese Medicine, Kunming, China Yunnan University of Chinese Medicine Kunming China; 2 College of Life Sciences, Hubei Engineering Research Center for Protection and Utilization of Special Biological Resources in the Hanjiang River Basin/Jianghan University, Wuhan 430056, China Jianghan University Wuhan China; 3 Key Laboratory of Soybean Disease and Pest Control (Ministry of Agriculture and Rural Affairs), Nanjing Agricultural University, Nanjing 210095 Jiangsu, China Nanjing Agricultural University Nanjing China; 4 School of Integrative Medicine, Mae Fah Luang University, 333 Moo 1, Thasud, Muang, Chiang Rai 57100, Thailand Mae Fah Luang University Chiang Rai Thailand

**Keywords:** Environmental stress response, epigenetic regulation, *Fusarium oxysporum*, genome-wide identification, histone deacetylase

## Abstract

Histone deacetylases (HDACs) are key epigenetic regulators governing chromatin structure and gene expression, playing critical roles in growth, development, virulence, and multi-stress resistance of plant-pathogenic fungi. Despite their importance, the HDAC gene family (FoHDACs) in *Fusarium oxysporum* remains poorly characterized. Through genome-wide analysis, we identified 11 *FoHDAC* genes, phylogenetically classified into three subfamilies: Class I (2 genes), Class II (2 genes), and SIR2 (7 genes). Subcellular localization predicted 6 in the nucleus, 3 in the cytoplasm, and 2 in mitochondria, indicating functional diversity across organelles. Structural analyses revealed conserved domains/motifs specific to each subfamily. Genes showed asymmetric distribution across 6 chromosomes with no recent duplication events. Promoter analysis identified 22 putative *cis*-elements, including antioxidant (ARE, as-1) and stress response elements (STRE), linking FoHDACs to development and environmental responses. Functional annotation highlighted putative roles in transcriptional regulation, macromolecular catabolism, and heterochromatin assembly beyond core HDAC activity. Molecular docking showed binding affinities < -5 kcal/mol with significant differences across subfamilies. RT-qPCR revealed stage-specific expression: 8 genes peaked in dormant conidia, were suppressed during germination, and recovered during growth/sporulation; 2 showed continuous activation, and 1 was sporulation-specific. Abiotic stresses induced stimulus-dependent regulation, e.g., 33.67-fold repression of *FoHST3* under salt stress and > 100-fold induction of *FoHOS3* under cold stress. Collectively, our findings reveal that FoHDACs exhibit substantial functional diversity, forming a sophisticated regulatory network mediating fungal development and environmental adaptation.

## Introduction

Histone deacetylases (HDACs) catalyze the removal of acetyl groups from acetylated lysine residues in histone N-terminal tails and globular domains, establishing transcriptionally repressive chromatin states (Curcio et al. 2024). As evolutionarily conserved epigenetic regulators spanning eukaryotes to prokaryotes, fungal HDACs are phylogenetically categorized into three classes based on yeast orthology: Zn^2^+-dependent Class I (RPD3 family), Class II (HDA1 family), and NAD+-dependent Class III (sirtuins)(Ueda et al. 2017). Class I HDACs (e.g., *HOS2*, *RPD3*) integrate an N-terminal deacetylase domain with a C-terminal regulatory region to orchestrate basal transcription and developmental processes (Zhang et al. 2023). Class II members (e.g., *HDA1*, *HOS3*) feature extended C-terminal domains and mediate environmental stress responses and metabolic plasticity (Hu et al. 2019). Class III sirtuins (e.g., *Sir2*, *HST1-4*) possess unique NAD+-dependent catalytic domains; yeast Sir2 exemplifies their role in coupling metabolic signaling to epigenetic silencing through telomeric, mating-type, and rDNA repression complexes (Gartenberg 2000; Hou et al. 2025).

Mounting evidence underscores the centrality of epigenetic regulation in host-pathogen interactions (Gómez-Díaz et al. 2012), yet its mechanistic underpinnings in phytopathogenic fungi remain inadequately explored. Critical insights reveal that Class I HDACs govern fungal growth and virulence—deletion causes severe growth defects with H4 hyperacetylation (Jiang et al. 2020), while *Magnaporthe oryzae MoRPD3* overexpression enhances conidiation but abolishes pathogenicity (Lee et al. 2021; Lin et al. 2021). These enzymes additionally modulate conidiation, blue-light perception, and oxidative stress adaptation (Estrada-Rivera et al. 2019). Class II HDACs, though non-essential for basal growth, are indispensable for host colonization, secondary metabolism suppression, and osmotic/oxidative stress tolerance (Tribus et al. 2005; Speckbacher et al. 2024). Class III sirtuins regulate asexual development, carbon/nitrogen utilization, and thermal/UV stress resistance (Cai et al. 2021); *Fusarium verticillioides FvSIRT5* and *FvSIR2* potentially deacetylate H3K9/K14/K27 to control conidiation and secondary metabolism (Yang et al. 2025).

*Fusarium oxysporum*—a devastating soil-borne pathogen infecting over 100 crops including tomato, banana, and cotton—causes vascular wilt diseases incurring billion-dollar annual losses (Alabouvette et al. 2009). Its pathogenic lifecycle hinges on stress adaptation mechanisms: During soil dormancy, chlamydospores employ HDAC-mediated chromatin silencing to sustain metabolic quiescence; root invasion requires countermeasures against host oxidative burst (Ding et al. 2010; Baker et al. 2013); vascular colonization demands hyperosmotic tolerance (Suwandi et al. 2018). Supporting this, *ΔHos2* mutants exhibit hypersensitivity to H_2_O_2_, SDS, and NaCl. Notably, *ΔHos2* shows increased sensitivity to chlorothalonil but not mancozeb or boscalid, while other HDAC/HAT mutants retain wild-type fungicide tolerance—implying HDAC-specific stress adaptation confers evolutionary advantages in pathogenicity (Ma et al. 2021). Despite these advances, systematic characterization of the *FoHDACs* gene family, including its genomic organization, molecular features, and stress-responsive regulatory dynamics, remains elusive.

To address this knowledge gap, we integrated multi-scale bioinformatics strategies: Phylogenetic reconstruction, gene structure and synteny analyses elucidated evolutionary relationships; protein physicochemical characterization, domain dissection, and *cis*-regulatory element screening defined molecular regulatory bases; molecular docking simulations decoded FoHDAC interactions with histone deacetylase inhibitors; RT-qPCR profiling delineated stage-specific and stress-inducible expression patterns. This comprehensive study establishes a foundation for understanding FoHDAC-mediated epigenetic regulation in *F. oxysporum* pathogenesis and environmental adaptation.

## Material and methods

### Sequence retrieval and comparative analysis

*FoHDAC* genes and proteins were identified through systematic searches of the *Fusarium oxysporum* Fo47 genome in NCBI (https://www.ncbi.nlm.nih.gov/) and UniProt (https://www.uniprot.org/) databases. Based on the genomic sequence data of *F. oxysporum* strain Fo47 (accession number GCF_013085055.1) obtained from the NCBI database and the conserved domain models for histone deacetylase (HDAC; Pfam: PF00850) and sirtuin (Pfam: PF02146) retrieved from the InterPro Pfam database (https://www.ebi.ac.uk/interpro/entry/pfam), HMMER was employed to identify putative *FoHDAC* gene sequences using a significance threshold of E-value < 1 × 10^-5^ and a minimum score of 80. Reference *HDAC* sequences from *Saccharomyces cerevisiae*, *Arabidopsis thaliana*, and *Homo sapiens* were obtained from the cited literature and used to construct a phylogenetic tree (Pandey et al. 2002). Protein sequences were aligned using MUSCLE in MEGA v7.0 with default parameters (Tamura et al. 2021), and phylogenetic trees were constructed via the neighbor-joining method (1,000 bootstrap replicates). Resultant trees were visualized using FigTree v1.4.5 and refined in Adobe Illustrator 2024. Three-dimensional structural alignments were conducted using the align command in PyMOL v2.5.2 for the purpose of validating the newly identified FoHDACs, measuring their evolutionary conservation and divergence, and visualizing whether the catalytic residues and motifs occupy the expected structural positions. Physicochemical properties (molecular weight, theoretical isoelectric point (pI), amino acid composition) were analyzed using ExPASy ProtParam. Subcellular localization was predicted via UniProt and CELLO v.2.5(Chou and Shen 2008; Duvaud et al. 2021).

### Sequence and structural characterization of FoHDACs

Gene structure features (intron/exon organization) were visualized using TBtools v2.003(Chen et al. 2020). Conserved domains were identified via NCBI-CDD and mapped onto predicted tertiary structures using PyMOL (Wang et al. 2023). Motif analysis was conducted using MEME Suite v5.5.2 (settings: maximum motifs = 10, width = 6-50 aa, https://meme-suite.org/meme/), with visualizations generated by TBtools and ggseqlogo package in R v4.3.1.

### Chromosomal localization and synteny analysis

Genome assemblies and annotation files for *F. oxysporum* (GCA_013085055.1), *F. graminearum* (GCA_000240135.3), *F. solani* (v2.0.60), and *M. oryzae* (MG8.60) were obtained from Ensembl Fungi. Chromosomal distributions and syntenic relationships were analyzed using MCScanX implemented in TBtools with default parameters (E-value: 0.001).

### *Cis*-regulatory element analysis

Promoter regions (2 kb upstream of translation start sites) were extracted using TBtools. Putative *cis*-regulatory elements were predicted using PlantCARE and functionally categorized (Lescot et al. 2002). Results were visualized via TBtools and ggplot2/tidyverse packages in R, with color schemes optimized using RColorBrewer.

### Functional annotation and molecular docking

Gene Ontology (GO) enrichment analysis was performed using STRING v12.0 (confidence score: 0.7)(Szklarczyk et al. 2023). Protein structures were modeled via SWISS-MODEL and AlphaFold2, with model quality assessed by QMEAN scores. Histone deacetylase (HDAC)-inhibitor complex Structures (PDB IDs: 8IJ0, 5I3L, 3O34, 5XTZ, 5VS7, 7EID, 3QZT) corresponding to acetylated histone substrates (H3K9ac, H3K14ac, H3K23ac, H3K27ac, H4K5ac, H4K8ac/H4K12ac, H4K16ac, respectively) were obtained from the RCSB Protein Data Bank. Protein preparation (deprotonation, solvation, energy minimization) used AutoDockTools v1.5.7. Molecular docking was executed in AutoDock Vina v1.2.3. Binding affinities ≤-5.0 kcal/mol indicated significant interactions, with results visualized in PyMOL+v2.6 and LigPlot+v2.2.5.

### Expression profiling under developmental stages and stress conditions

The *F. oxysporum* strain was cultured on potato dextrose agar (PDA) at 25 °C (Liao et al. 2024). Spore suspensions containing 1 × 10^7^ conidia/mL in 0.01% Tween-80 were inoculated into YEPD liquid broth and harvested across four defined developmental stages: dormant conidia at 0 h (G1), germinating conidia at 8 h post-inoculation (G2), actively growing mycelia at 24 h (G3), and sporulating hyphae at 72 h (G4). For stress treatments, mycelial mats (1 g wet weight) were subjected to osmotic stress with 1 M NaCl or 1 M sorbitol, oxidative stress with 10 mM H_2_O_2_, fungicide exposure to 1 mM hymexazol or 1 mM fludioxonil, and thermal stress at 4 °C or 37 °C using sterile water-treated samples maintained at 28 °C as controls. All treatments were conducted in shaking incubators (180 rpm) for a standardized duration of 24 h. Three biological replicates per treatment group were immediately flash-frozen in liquid nitrogen post-harvest for downstream analysis.

Total RNA was extracted using TRIzol^®^ (Invitrogen), reverse-transcribed with PrimeScript™ RT reagent kit (Takara), and quantified by RT-qPCR on QuantStudio 5 (Applied Biosystems) with SYBR^®^ Green master mix. A set of specific primers targeting all 11 identified FoHDAC genes was designed in silico using Primer-BLAST to facilitate their expression profiling across different developmental stages and stress conditions. The specificity of these primer pairs (Suppl. material 1: table SS1) was further experimentally validated by melt curve analysis. The *β-tubulin* gene (QTUB) served as the reference (Liao et al. 2024). Relative expression was calculated using the 2^-ΔΔCt^ method with three technical replicates. Statistical significance was assessed by ANOVA with Tukey’s HSD (*p < 0.05*).

## Results

### Identification and phylogenetic analysis of FoHDACs

**Figure 1.**
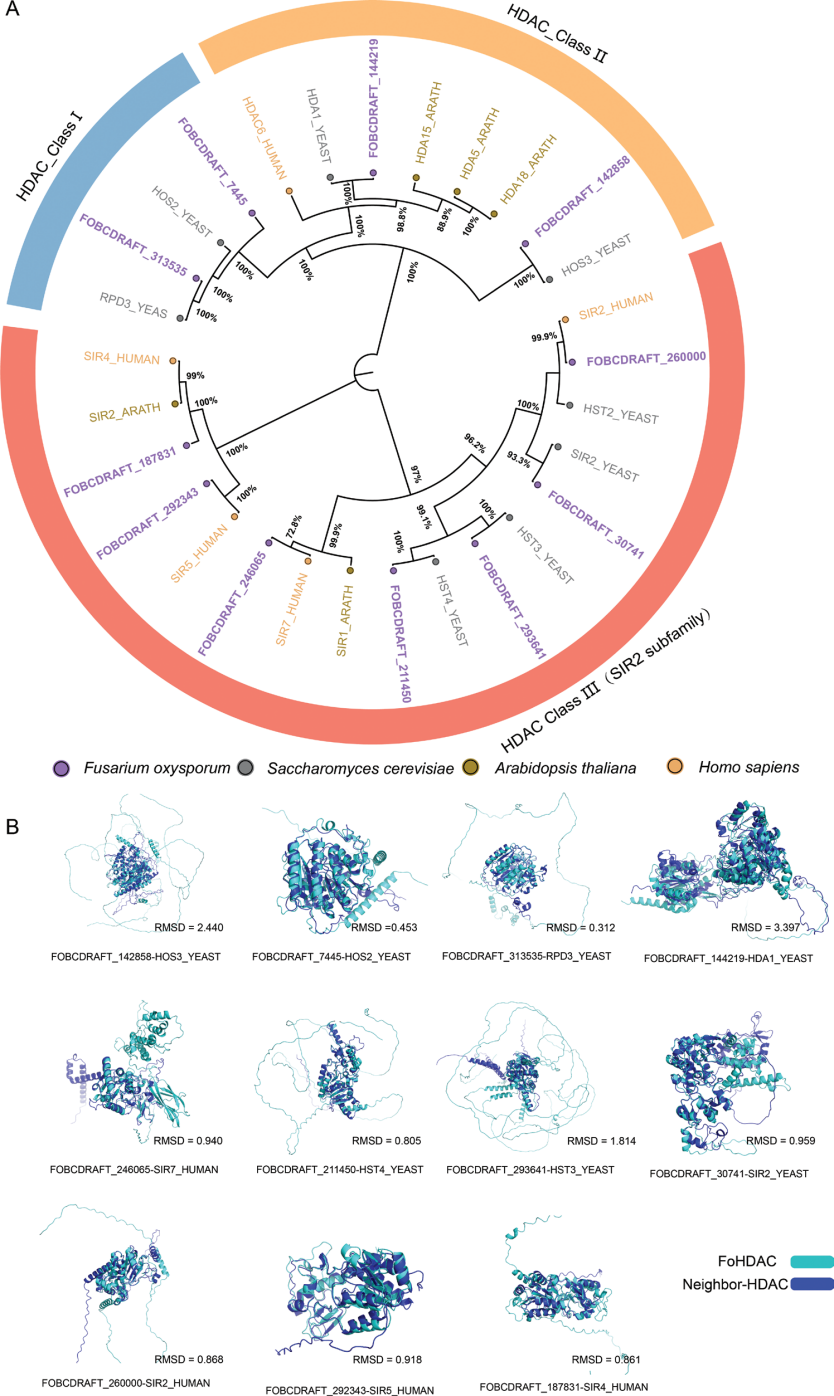
Phylogenetic and structural analysis of histone deacetylases in *F. oxysporum*. **A** Neighbor-joining phylogenetic tree constructed from aligned FoHDAC domain amino acid sequences using MEGA11 (1,000 bootstrap replicates). The topology delineates evolutionary relationships among HDACs from *F. oxysporum* (purple), *S. cerevisiae* (dark gray), *A. thaliana* (gold), and *H. sapiens* (orange). **B** Structural superpositions of FoHDACs with phylogenetically adjacent orthologs, generated in PyMOL. Root Mean Square Deviation (RMSD) values (Å) quantify backbone atom positional variances, with lower values indicating higher structural conservation.

Comparative analysis of HDAC sequences from *F. oxysporum*, *S. cerevisiae*, *A. thaliana*, and *H. sapiens* enabled systematic classification of the FoHDAC gene family. ClustalW and neighbor-joining phylogenetic reconstructions (Fig. 1A) resolved HDACs into three evolutionarily conserved classes: HDAC Class I, HDAC Class II, and HDAC Class III (SIR2 subfamily). Among the 11 identified FoHDAC genes (Fig. 1A, Table 1), phylogenetic distribution revealed dominance of the SIR2 subfamily (7 genes), with Class I and Class II each containing two members.

**Table 1.** Physicochemical properties of FoHDACs gene family members.

Class	Gene Name	Locus_tag^a^	Protein ID^b^	Gene length (bp)	Protein length (AA)	Molecular Weight (Da)	Theoretical pI	Instability Index	Aliphatic Index	Grand Average of Hydropathicity	Prediction of Subcellular Localisation
HDAC Class I	*FoHOS2*	FOBCDRAFT_7445	XP_031043728.1	1767	499	56120.16	5.46	35.29	78.4	-0.453	Cytoplasm
	*FoRPD3*	FOBCDRAFT_313535	XP_031041597.2	2799	650	72115.33	4.53	39.87	59.46	-0.793	Nucleus
HDAC Class Ⅱ	*FoHOS3*	FOBCDRAFT_142858	XP_059465600.1	3546	1087	117766.53	9.42	59.36	66.96	-0.656	Nucleus
	*FoClr3*	FOBCDRAFT_144219	XP_031033864.2	2429	736	82777.03	5.37	40.39	80.29	-0.361	Cytoplasm
HDAC Class ⅡI (SIR2 subfamily)	*FoSIR7*	FOBCDRAFT_246065	XP_059468203.1	2,500	626	68510.51	5.52	47.55	79.78	-0.283	Nucleus
	*FoHST4*	FOBCDRAFT_211450	XP_031042553.2	2639	624	68326.29	9.5	46.17	66.11	-0.747	Nucleus
	*FoHST3*	FOBCDRAFT_293641	XP_031040443.2	3103	953	104927.26	7.19	60.65	74.18	-0.617	Nucleus
	*FoSIR2*	FOBCDRAFT_30741	XP_031044680.2	1940	487	54578.07	6.88	45.09	82.28	-0.485	Nucleus
	*FoHST2*	FOBCDRAFT_260000	XP_031044378.2	1753	437	47798.75	4.8	43.59	74.35	-0.542	Cytoplasm
	*FoSIR5*	FOBCDRAFT_292343	XP_031044252.3	999	298	32799.45	5.54	40.38	81.61	-0.301	Mitochondrion
	*FoSIR4*	FOBCDRAFT_187831	XP_031035749.3	1657	449	48764.98	8.71	37.94	91.31	-0.115	Mitochondrion

a: This locus_tag is derived from the *F. oxysporum* Fo47 genome assembly GCF_013085055.1. b: All protein IDs are computational prediction models (XP_) derived from the *F. oxysporum* Fo47 RefSeq genome assembly GCF_013085055.1.

Structural conservation was quantitatively assessed through tertiary structure alignments using PyMOL. Root-mean-square deviation (RMSD) values between FoHDACs and their orthologs ranged from 0.312 to 3.397 Å (Fig. 1B), indicating divergent evolutionary trajectories. Notably, *FOBCDRAFT_313535* exhibited exceptional structural similarity to yeast *RPD3* (RMSD = 0.312 Å), with conserved catalytic pocket geometry validating phylogenetic clustering accuracy. In contrast, *FOBCDRAFT_144219* displayed higher structural divergence from yeast *HDA1* (RMSD = 3.397 Å), yet retained critical active-site residue conservation (Suppl. material 1: figs S1, S2). This pattern is consistent with structural plasticity, which may facilitate functional adaptation to environmental pressures. This suggests potential structural plasticity facilitating functional adaptation to environmental pressures (Kiefl et al. 2023). Based on integrated phylogenetic-structural evidence, we propose a standardized nomenclature for FoHDAC genes (Table 1).

### Physicochemical profiling and subcellular localization of FoHDACs

Molecular weight, instability index, and hydrophobicity constitute fundamental determinants of protein primary structure that influence functional competence. Systematic characterization of FoHDACs using ExPASy ProtParam revealed substantial variation in amino acid lengths (298–1087 residues), corresponding to molecular masses spanning 32.8–117.8 kDa. pI analysis classified seven proteins (including *FoHDAC3* and *FoRPD3*) as acidic (pI < 7.0), three (e.g., *FoHOS3*) as basic (pI > 7.0), and *FoSIR2* as near-neutral (pI = 6.88). Stability assessment indicated that *FoHDAC3*, *FoRPD3*, and *FoSIR4* exhibited high conformational stability (instability index < 40), whereas other members displayed moderate instability. Determining the subcellular localization of FoHDACs proteins will facilitate a better understanding of their molecular functions. According to the prediction of FoHDACs subcellular localization, FoHOS3, FoRPD3, FoSIR7, FoHST4, FoHST3, and FoSIR2 are localized to the nucleus; FoHDAC3, FoClr3, and FoHST2 are localized to the cytoplasm; while FoSIR5 and FoSIR4 are localized to the mitochondrion (Table 1).

All FoHDACs demonstrated hydrophilic properties (grand average of hydropathy, GRAVY < 0), with a significant negative correlation observed between hydrophilicity and aliphatic index (R² = 0.878, p < 0.01, Table 1). This inverse relationship suggests that reduced non-polar amino acid content enhances solubility, potentially facilitating protein-nucleic acid interactions. This integrated profiling establishes a structural basis for understanding functional diversification within the FoHDAC family.

### Structural and motif characterization of FoHDACs

**Figure 2.**
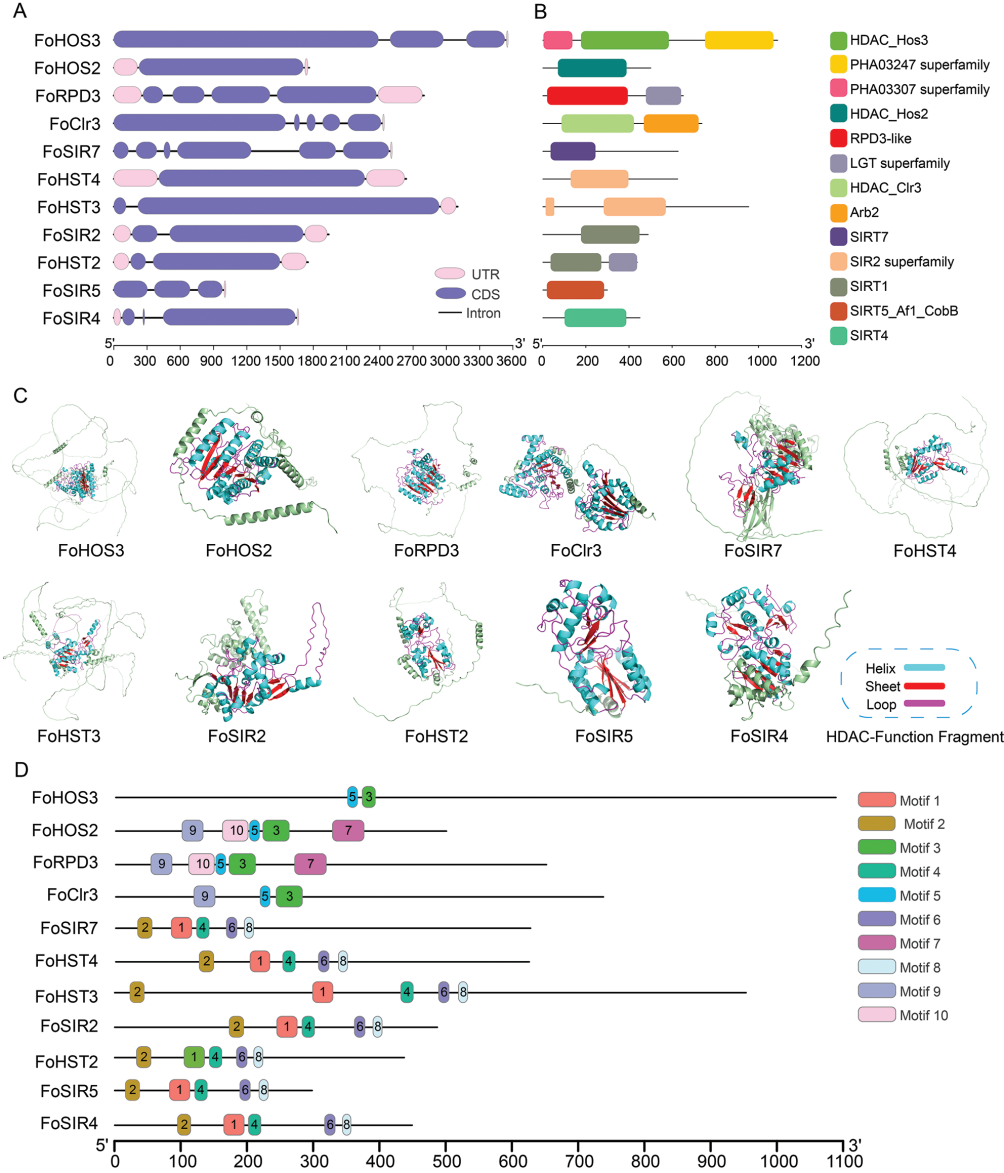
Structural architecture and conserved signatures of *FoHDAC* family members. **A** Intron-exon organization illustrating gene structural diversity. **B** Conserved domain annotation predicted by NCBI Conserved Domain Database with E-value ≤ 0.01. **C** Catalytic pocket geometry within tertiary structures visualized through PyMOL v2.5.2 (surface representation). **D** Distribution of ten conserved motifs identified by MEME Suite v5.5.2 (width: 6-50 aa; E-value < 0.01), color-coded according to motif classification.

Comprehensive characterization of FoHDAC gene architectures revealed significant structural diversity across the family. All members feature 4–7 introns exhibiting substantial variation in genomic positioning, phase conservation, and length distribution (Fig. 2A). Domain architecture analysis further demonstrated that while most FoHDACs retain the conserved catalytic core, distinctive structural features emerge in specific members (Fig. 2B). Notably, FoHOS3 harbors auxiliary viral transcriptional regulator domains (PHA03307/PHA03247 superfamilies, Suppl. material 1: table S2) that coordinate HDAC-mediated chromatin regulation in viral systems (Lomonte et al. 2004), suggesting potential functional conservation in fungal epigenetics. Concurrently, FoRPD3 and FoHST2 contain lipoprotein diacylglycerol transferase domains (LGT superfamily, Suppl. material 1: table S2) with dual enzymatic capabilities that may modulate acetyl-CoA substrate availability (Zan et al. 2019).

Tertiary structure modeling confirmed the catalytic sites reside within evolutionarily conserved cores critical for structural integrity (Fig. 2C). Intriguingly, although essential catalytic residues display non-contiguous primary sequence distribution, they converge into spatially organized active sites through precise α-helix/β-sheet folding topologies - a structural prerequisite for enzymatic function. Supporting these findings, motif analysis identified ten conserved signatures with distinct subfamily associations (Fig. 2D, Suppl. material 1: fig. S3). Class I/II HDACs universally share Motif 3 and 5, while Motif 9 exhibits near-ubiquitous distribution excluding *FoHOS3*. The complete sequence identity of Motif 7 and 10 in *FoHDAC3* and *FoRPD3* aligns with their phylogenetic clustering and implies functional redundancy. SIR2 subfamily members display particularly deep evolutionary conservation through universal retention of Motif 1/2/4/6/8. Functional analysis of these motifs revealed that Motif 1 and Motif 3 are associated with distinct molecular functions corresponding to two types of histone deacetylase activities (Suppl. material 1: table S3), suggesting their potential role as putative catalytic cores in *FoHDACs*. In contrast, Motif 4 remains functionally unannotated and warrants further investigation.

### Chromosomal distribution and syntenic conservation of FoHDACs

**Figure 3.**
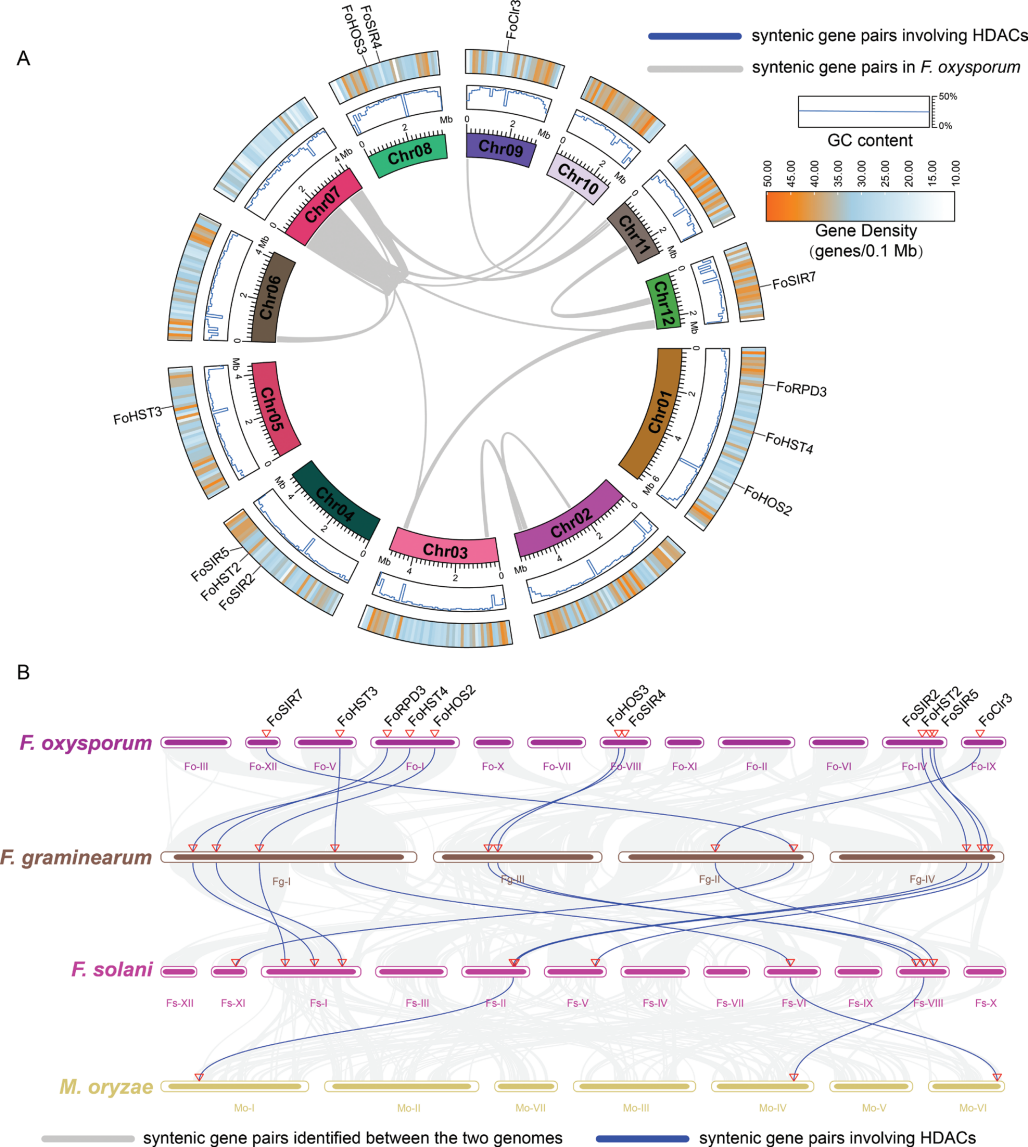
Genomic architecture and evolutionary conservation of *FoHDAC* genes. **A** Chromosomal distribution in *F. oxysporum* is visualized through a Circos plot featuring chromosome identifiers and lengths (Mb) in the innermost ring, GC content (%) in the middle ring, and gene density (genes/0.1 Mb) with FoHDAC positions in the outermost ring. **B** Comparative synteny analysis across *F. graminearum*, *F. solani*, and *Magnaporthe oryzae* delineates conserved genomic contexts wherein gray lines connect syntenic blocks and blue lines highlight orthologous HDAC gene pairs.

Comprehensive genomic analysis revealed distinct organizational patterns and evolutionary trajectories of the FoHDAC gene family. Chromosomal mapping demonstrated non-uniform distribution across the *F. oxysporum* genome (Fig. 3A): Chromosomes 1 and 4 harbored three genes each (*FoHOS2*/*FoHST4*/*FoRPD3* and *FoSIR5*/*FoHST2*/*FoSIR2*), chromosome 8 contained two genes (*FoSIR4*/*FoHOS3*), while chromosomes 5 (*FoHST3*) and 12 (*FoSIR7*) possessed single loci. Notably, six chromosomes (2, 3, 6, 7, 10, 11) lacked FoHDAC genes entirely. Gene density profiling revealed that the majority of members were located in low-to-medium density regions, consistent with their broad involvement in transcriptional regulation. In contrast, FoSIR7 and FoRPD3 were notably observed to reside in high-density regions, suggesting functional divergence from other family members, potentially specifically regulating heterochromatin-related functions (Fig. 3A).

Synteny analysis excluded recent segmental duplications as drivers of family expansion (Fig. 3A). Comparative genomics across *Fusarium* species identified strict orthologous conservation: All 11 *FoHDACs* maintained one-to-one orthology with *F. graminearum* and *F. solani* homologs (Fig. 3B). However, only three SIR2-class genes (*FoHST3*, *FoSIR2*, *FoSIR4*) showed cross-family orthology with *M. oryzae*. This exceptional conservation of SIR2 members across taxonomic boundaries implies their essential role in core epigenetic machinery, potentially enabling fungal adaptation to host-specific niches and environmental stressors.

### *Cis*-regulatory element profiling in FoHDAC promoters

**Figure 4.**
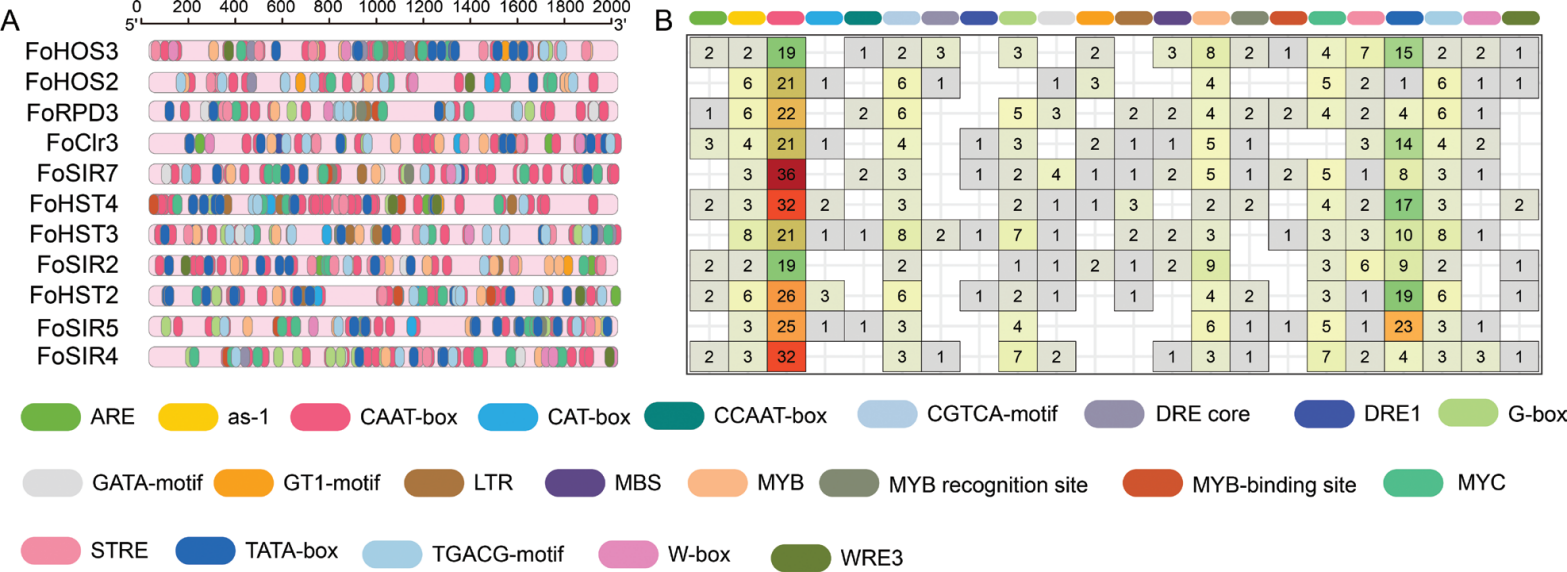
Characterization of *cis*-regulatory elements in *FoHDAC* promoter regions. **A** Distribution profile of *cis*-element categories within 2-kb upstream promoter sequences of *FoHDAC* genes. **B** Quantitative analysis of *cis*-regulatory elements, with color-coded boxes representing distinct functional categories.

Histone deacetylases orchestrate plant developmental programs and abiotic stress adaptation (Wang et al. 2024), yet their regulatory architecture in phytopathogenic fungi remains underexplored. We systematically characterized *cis*-regulatory elements within 2-kb promoter regions of FoHDAC genes, revealing 22 functionally annotated motifs (Fig. 4A). Core transcriptional regulatory elements (Laloum et al. 2013; Chen et al. 2023) (e.g., CAAT-box, TATA-box) co-occur with stress-responsive *cis*-regulatory motifs, including antioxidant response elements (Garretón et al. 2002; Xia et al. 2016) (ARE, as-1) and stress response elements (Schüller et al. 1994) (STRE) (Fig. 4B).

Non-biotic stress modules dominate the regulatory landscape, with dehydration-responsive elements (DRE)(Agarwal et al. 2017), MYB/MYC recognition site (Abe et al. 2003), and W-box (Liu et al. 2016) ubiquitously distributed (Fig. 4B). This configuration implicates FoHDACs in drought and salinity adaptation. Crucially, universal conservation of as-1 motif across all members signifies essential oxidative stress mitigation roles. The pervasive occurrence of STRE elements further corroborates transcriptional reprogramming during thermal/osmotic challenges. Notably, WRE elements exhibit selective enrichment in six genes (e.g., *FoHOS2*, *FoHST4*, etc.), suggesting spatiotemporal regulatory specialization for developmental coordination (Fig. 4B). Complementary stress sensors (GACG-motif, LTR, G-box) expand environmental signal integration capacity (Lamers et al. 2020). Mechanistically, stress-induced second messengers (Ca²+, ROS) activate transcription factors that target cognate cis-motifs—MYB proteins binding MYB-sites, WRKY factors engaging W-boxes—thereby modulating FoHDAC expression. This transcriptional circuitry enables rapid epigenetic reconfiguration through chromatin remodeling, positioning *FoHDACs* as central adaptors in fungal environmental sensing.

### Functional diversification of FoHDACs

**Figure 5.**
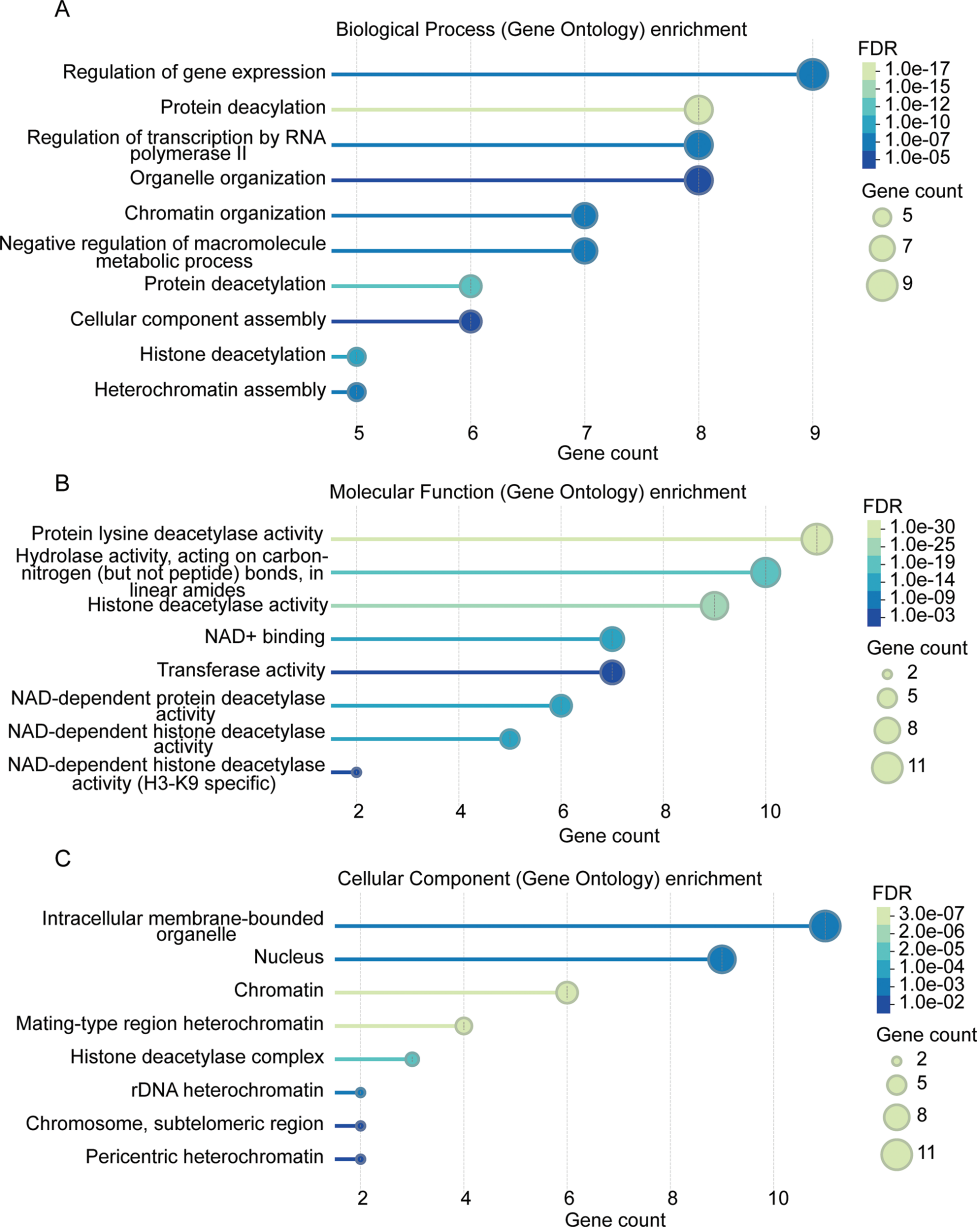
Gene Ontology enrichment profiling of FoHDAC genes. **A** Top 10 significantly enriched biological processes. **B** Top 8 molecular functions. **C** Top 8 cellular components. Horizontal axes indicate gene counts per category; vertical axes display GO term descriptions.

Integrated bioinformatic and computational analyses delineated functional specialization within the *F. oxysporum* histone deacetylase family. Gene Ontology (GO) enrichment revealed roles beyond core deacetylation, including transcriptional regulation, macromolecular catabolism, and heterochromatin assembly (Fig. 5, Suppl. material 1: table S4). Notably, *FoHST4*, *FoHST3*, and *FoClr3* participate in cellular component organization (Suppl. material 1: table S4), implicating specialized functions in sporulation and cell wall biogenesis (Du et al. 2022). While all FoHDACs displayed lysine deacetylase activity (Fig. 5B), *FoSIR2/7* showed exclusive specificity for H3K9 deacetylation (Suppl. material 1: table S4). Strikingly, *FoHST4*, *FoHST3*, and *FoSIR2* exhibited bifunctionality, possessing intrinsic histone acyltransferase activity (Suppl. material 1: table S4). Cellular compartmentalization analysis localized all FoHDACs to membrane-bound organelles, with nine members (e.g., *FoHST4*, *FoHOS3*, etc.) specifically nuclear-localized (Fig. 5C, Suppl. material 1: table S4). Crucially, *FoClr3*, *FoHOS2*, and *FoRPD3* were annotated as core components of HDAC complexes, suggesting enhanced regulatory precision through macromolecular assembly.

### Substrate specificity of FoHDACs

**Figure 6.**
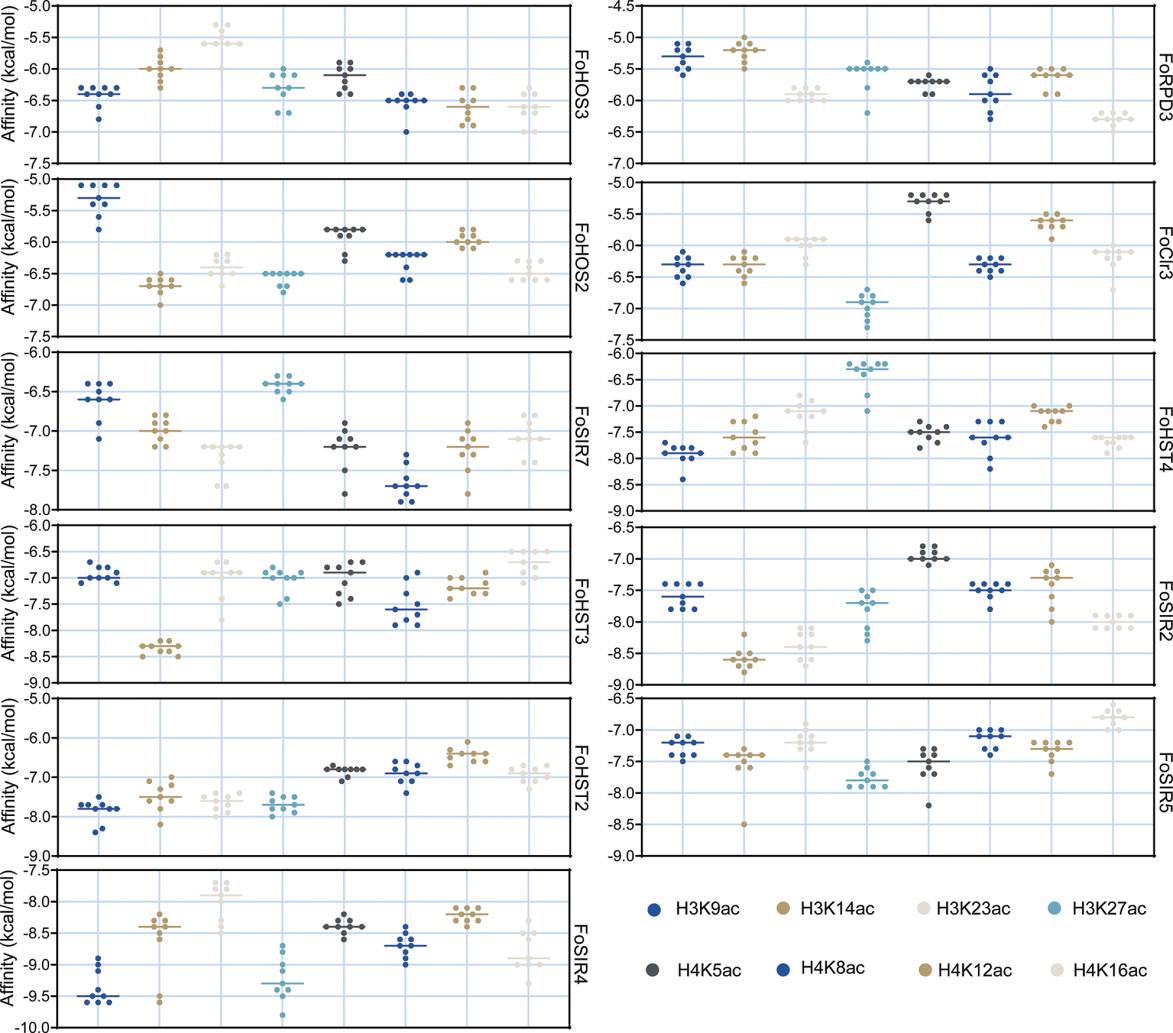
Molecular docking analysis of FoHDAC interactions with histone deacetylase modification sites. Binding affinities (kcal/mol) are shown for nine representative FoHDAC-substrate complexes. For each enzyme, the binding pose exhibiting maximal absolute affinity was selected for detailed interaction analysis. Structural visualizations were generated in PyMOL v2.5.2 (surface representation).

Molecular docking simulations targeting eight histone acetylation marks—including H3K9ac and H3K14ac—demonstrated robust binding affinities across all FoHDACs (Affinity ≤ -5 kcal/mol; Fig. 6, Suppl. material 1: fig. S4), confirming their biological relevance in epigenetic regulation. Phylogenetic-structural clustering resolved two functionally distinct subfamilies (Suppl. material 1: figs S5–S10). HDAC-class members employ conserved His/Asp catalytic dyads while exhibiting specialized substrate preferences: FoHOS3 selectively engages H4K16ac whereas FoClr3 targets multiple sites including H3K27ac and H4K12ac. Contrastingly, sirtuin-class proteins display broader substrate recognition patterns through complex catalytic pocket architectures; FoHST3 binds all tested sites with H3K14ac predilection, FoSIR2 shows high affinity for H3K14ac/H3K23ac, and FoSIR5 recognizes five modification sites including H3K9ac, while FoSIR7 demonstrates attenuated binding activity potentially indicating non-catalytic regulatory functions.

### Developmental and stress-responsive expression dynamics of *FoHDACs*

**Figure 7.**
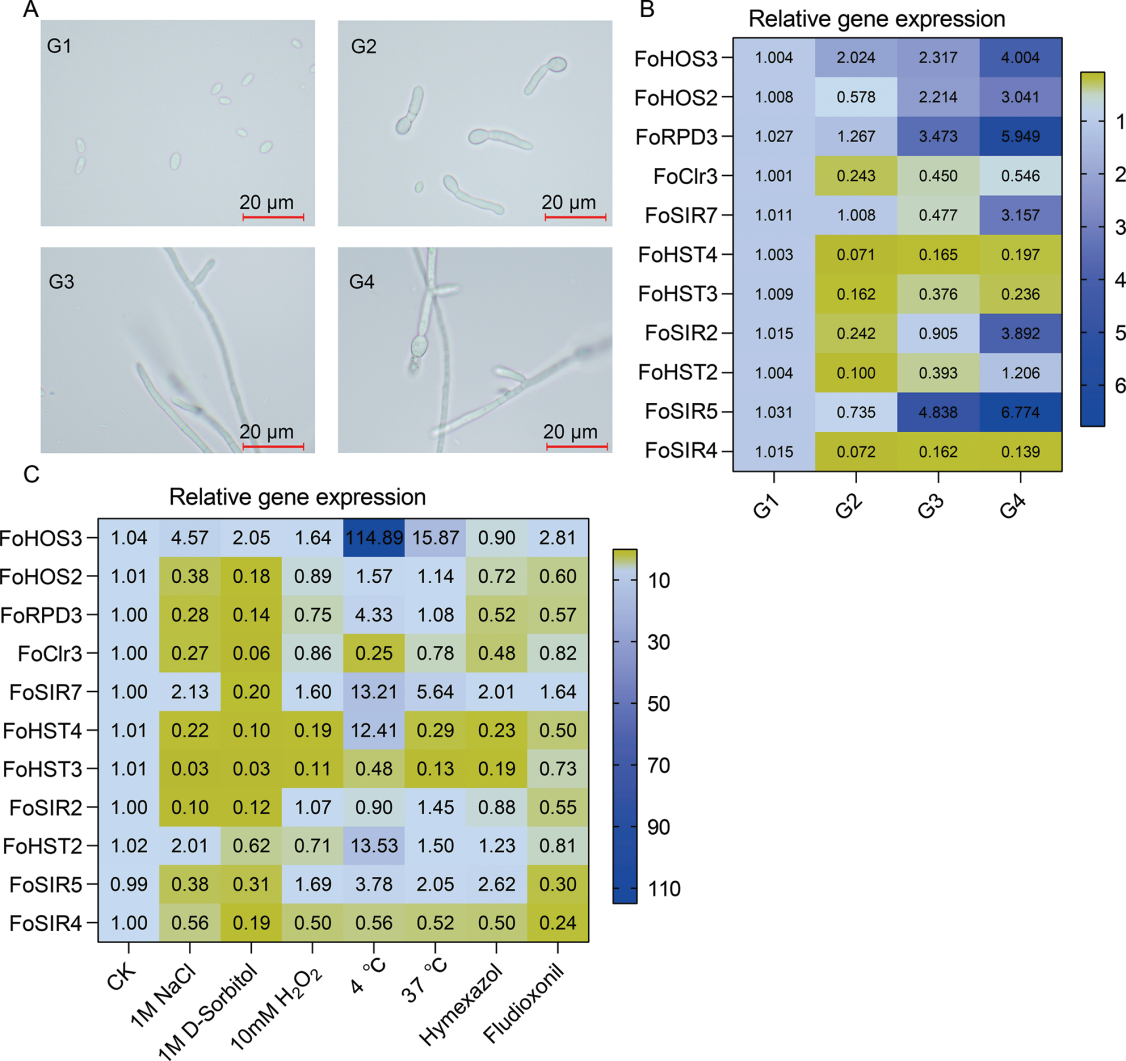
Expression profiling of FoHDAC genes across developmental stages and stress conditions. **A** Morphological transitions of *F. oxysporum*: sporomorphic phase (G1), spore germination (G2), mycelial growth (G3), and sporulation (G4). **B** Stage-specific transcript abundance relative to G1 baseline. (C) Differential expression under abiotic stressors (salt, osmotic, oxidative, cold, heat) and fungicide treatments (hymexazol, fludioxonil).

Comprehensive expression profiling via RT-qPCR revealed stage-specific regulation of *FoHDAC* genes during *F. oxysporum* development. Eight genes (including *FoHOS2*, *FoClr3*, *FoHST4*, *FoHST3*, *FoSIR2*, *FoHST2*, *FoSIR5 and FoSIR4*) exhibited maximal transcript levels in sporomorphic phase (G1), underwent substantial suppression during spore germination (G2), and displayed progressive recovery through mycelial growth (G3) culminating in sporulation (G4) (Fig. 7A, B). In contrast, *FoHOS3* and *FoRPD3* manifested continuous transcriptional activation across developmental transitions, while *FoSIR7* demonstrated exclusive sporulation-phase induction (Fig. 7B). These phased expression dynamics suggest specialized epigenetic coordination of morphogenetic progression.

Under abiotic stresses, *FoHDACs* exhibited stimulus-dependent modulation (Fig. 7C). Salt stress (1 M NaCl) broadly repressed transcription, most dramatically in *FoHST3* with 33.67-fold suppression, though *FoHOS3* and *FoSIR7* showed moderate induction. Osmotic challenge (1 M sorbitol) selectively activated *FoHOS3* expression. Oxidative conditions (10 mM H_2_O_2_) significantly elevated *FoHOS3*, *FoSIR7*, and *FoSIR5* transcripts while suppressing *FoHST4*. Thermal extremes induced dichotomous responses: cold stress (4 °C) triggered extreme *FoHOS3* induction exceeding 100-fold, with coordinated activation of *FoHOS2*, *FoRPD3*, and *FoSIR7*, whereas heat stress (37 °C) predominantly upregulated targets including *FoHOS3* (15.26-fold) despite repressing *FoHST4* and *FoHST3*. Fungicide exposure demonstrated compound-specific effects, with fludioxonil activating *FoHOS3* and *FoSIR7*, and hymexazol inducing *FoSIR7*, *FoHST2*, and *FoSIR5*.

This combinatorial regulation designates *FoHOS3* as a master stress integrator, consistently induced across osmotic, oxidative, thermal, and chemical challenges. *FoSIR7* functions as a broad-spectrum responder, while *FoHST3* and *FoHST4* typically undergo stress-associated repression. Such stimulus-specific reprogramming establishes FoHDACs as central architects of epigenetic adaptation.

## Discussion

Studies have shown that histone acetyltransferases not only regulate key biological processes of fungi such as sporulation, germination, and pathogenicity, but also participate in plant defense responses (Kong et al. 2018). However, current research on the functions of histone deacetylases (HDACs) has mainly focused on model organisms like yeast and *A. thaliana* (Yan et al. 2023), with relatively few systematic studies on HDACs in *F. oxysporum* (Zhang et al. 2024a). By integrating bioinformatics, 11 *FoHDACs* genes encoding HDAC domains were identified in *F. oxysporum*. Phylogenetic analysis (Fig. 1A) clearly classified them into three subfamilies: NAD+-dependent Sirtuin clade (7 members) alongside Zn²+-dependent Rpd3/Hda1-type HDACs (Class I/II, 2 members each). Domain analysis confirmed the reliability of this classification (Fig. 2B). Motif characteristics showed that the Class I/II subfamilies share conserved motif 3 and 5, while the SIR2 subfamily contains 5 unique conserved motifs (Fig. 2D). This gene family exhibits conservation within subfamilies and specificity between subfamilies in terms of conserved domains and motifs. The fundamental similarity of these molecular features to the established architecture of HDACs in species like *A. thaliana*, yeast, and humans suggests a high degree of functional stability for this gene family during evolution (Hou et al. 2021; Yang et al. 2023).

Compared with HDACs in other fungi, *FoHDACs* demonstrate both evolutionary conservation and lineage-specific characteristics. For instance, the number and composition of HDACs in *F. oxysporum* are largely consistent with those reported in the filamentous fungus *Fusarium verticillioides* and *M. oryzae* (Yu et al. 2024), where similar Class I/II and SIR2 members have been identified. In contrast, other significant fungal pathogens such as *Alternaria alternata*, and *Candida albicans* have been reported to contain only 6–8 HDAC members (Ma et al. 2021; Zhao and Rusche 2021). Notably, the SIR2 subfamily exhibits a larger number of members compared to the classical Zn^2+^dependent HDAC subfamilies, suggesting that NAD^+^-dependent deacetylases may play more diverse and specialized roles in the adaptation of *Fusarium* pathogens to varied hosts or environmental niches (Cai et al. 2024; Ren et al. 2024). Structurally, the conserved motifs identified in FoHDACs—such as motif 3 and 5 in Class I/II HDACs—are also present in orthologs from *F. verticillioides*, implying conserved catalytic or regulatory functions (Lin et al. 2021; Yu et al. 2024). On the other hand, the unique motifs within the FoHDAC SIR2 subfamily may underlie functional specialization, potentially influencing processes such as secondary metabolism or host invasion. Supporting this notion, studies in *Colletotrichum gloeosporioides* have demonstrated that the histone deacetylase HOS2 regulates appressorium formation and melanin biosynthesis (Liu et al. 2022). These findings collectively suggest that certain *FoHDACs* may similarly govern pathogenicity-related traits in *F. oxysporum*.

Studies have shown that HDACs are involved in plant development and abiotic stress responses (Yuan et al. 2020; Sun et al. 2021), but their regulatory mechanisms in plant pathogenic fungi remain unclear. This study conducted a systematic analysis of the promoter regions of *FoHDACs*, revealing a complex composition of *cis*-acting elements (Fig. 4). A total of 22 types of elements were identified, including core abiotic stress-responsive elements such as STRE, ARE, and DRE. The widespread distribution of these elements suggests that the FoHDAC family may be broadly involved in the epigenetic regulatory network enabling *F. oxysporum* to cope with environmental stresses. Notably, the promoter region of *FoHOS3* is enriched with multiple ARE and DRE elements, and its significant upregulation under oxidative stress (H_2_O_2_) and osmotic stress (NaCl, D-Sorbitol) closely correlates with this structural feature (Fig. 7C). This finding is consistent with the function of DRE and ABRE elements in the soybean *GmNCED5* gene (Kun et al. 2021). Additionally, *FoHST2*, *FoSIR7*, and *FoHOS3* showed 2–4 fold upregulation under salt stress (Fig. 7C), a response pattern similar to that of the rice HDAC*OsHDA706*(Liu et al. 2023). The upregulation of these genes under low-temperature stress may be associated with STRE and MBS elements in their promoters (Cui et al. 2024). Beyond stress response, development-related elements such as GATA-motif and CCAAT-box were also identified in the promoters (Fig. 4B).

Correspondingly, during mycelial growth (G1) and sporulation (G4) stages, the expression of multiple Class I/II HDACs (e.g., *FoHOS3*, *FoRPD3*) and SIR2 members (e.g., *FoSIR5*, *FoSIR7*) showed significant differences (Fig. 7B), indicating their potential roles in regulating hyphal development and sporulation. Particularly noteworthy is the selective distribution of the WRE3 element in the promoter regions of specific genes (e.g., *FoHOS2*, *FoHST4*), with these genes exhibiting similar expression trends during growth and development, suggesting their simultaneous involvement in both stress response and developmental transitions (Rahman et al. 2021).

Regarding fungicide stress, the upregulation of *FoHST2* and *FoSIR7* under hymexazol stress (Figs 4, 7C) may be related to the activation of oxidative stress-responsive elements such as as-1 in their promoters by ROS signals (Garretón et al. 2002). Conversely, the induced expression of *FoHST3* and *FoHST2* under fludioxonil stress is likely mediated by STRE elements in their promoter regions (Figs 4, 7C), consistent with the activation mechanism of the HOG-MAPK pathway (Schüller et al. 1994). This study has certain limitations. The absence of significant expression changes in some genes containing stress-responsive elements (e.g., *FoSIR2*) under corresponding stresses indicates that promoter structure is not the sole determinant of gene response (Figs 4, 7C). This phenomenon may result from factors such as epigenetic silencing effects (Zhang et al. 2024b), or insufficient transcription factor activity (Estruch 2000). Future research should integrate chromatin accessibility (ATAC-seq) and histone modification (ChIP-seq) analyses to elucidate the finer transcriptional regulatory mechanisms of FoHDACs in stress responses from multiple dimensions, thereby improving our understanding of their functional networks.

Histone acetylation plays a core regulatory role in key biological processes of filamentous fungi, such as morphogenesis, conidiospore development, and secondary metabolism. HDACs regulate gene expression by specifically removing acetyl groups from lysine residues at the N-terminus of histones (H2A/H2B/H3/H4). In this study, molecular docking simulations (Fig. 6) were used to analyze the binding properties of the HDAC family (FoHDACs) in *F. oxysporum*. The results verified known regulatory patterns: ScHOS3 preferentially acts on H4K8ac (Carmen et al. 1999); ScHOS2/ScClr3 target the deacetylation of H3(Wang et al. 2002; Liu et al. 2022); AnRPD3 can modify both H3 and H4(Tribus et al. 2010). Among SIR2 homologs, ScHST2 of *Saccharomyces cerevisiae* recognizes H3K9ac/H4K16ac (Imai et al. 2000); MrHST3/4 act on H3K56ac (Hu et al. 2023; Cai et al. 2024); while HsSIR4/5/7 exhibit low acetylation modification activity and tend to be involved in non-classical metabolic regulation, such as glutamine metabolism (Tomaselli et al. 2020), lysine desuccinylation (Park et al. 2013), and ribosome biogenesis (Sirri et al. 2019). These findings offer a novel perspective for dissecting the epigenetic regulatory network of *F. oxysporum* and provide a critical theoretical foundation for the development of next-generation fungicides targeting HDAC family members.

This study systematically reveals the epigenetic regulatory network of FoHDACs in *F. oxysporum*, providing multi-dimensional targets for disease control ranging from “life cycle intervention” to “stress response inhibition”. Future research needs to break through the bottleneck of functional verification through technological innovation, combine structural biology with translational studies, and promote the transformation of HDAC-targeted precision plant protection strategies from theory to application. This will provide a new paradigm for addressing fungal diseases and ensuring agricultural ecological security.

## Conclusion

This study provides the first systematic characterization of *F. oxysporum*’s 11-member HDAC family (*FoHDACs*). We reveal pathogen-specific evolutionary innovations within conserved structures and a unique genomic organization enabling functional diversification through specialization rather than gene duplication. FoHDACs serve as central hubs for environmental sensing, integrating stress signals via complex promoter *cis*-element networks and subfamily-divergent substrate recognition strategies. Functionally, they dynamically coordinate fungal adaptation through stress defense and developmental regulation. Crucially, we identify a three-phase epigenetic mechanism: dormancy (*FoHOS2/FoClr3*), virulence activation, and colonization (*FoSIR7*), forming the core regulator of pathogenic progression. These findings establish FoHDACs as high-value targets for designing precision antifungal inhibitors with minimal off-target effects, offering sustainable strategies to disrupt virulence in climate-stressed agriculture.

## Additional information

### Use of AI

No use of AI was reported.

### Adherence to national and international regulations

All the fungal strains used in this study have been legally obtained, respecting the Convention on Biological Diversity (Rio Convention).

### Data availability

All of the data that support the findings of this study are available in the main text or Supplementary Information.

## Supplementary materials

Supplementary material 1 Additional information Data type docx Explanation note **fig. S1**. Sequence alignment of HDAC class subfamily members. **fig. S2**. Sequence alignment of SIR2 class subfamily members. **fig. S3**. FoHDAC amino acid frequency of 10 conserved motifs. **fig. S4**. Three-dimensional structural visualization of histone acetylation modifications in the FoHDAC catalytic domain using PyMOL v2.5. **fig. S5**. Two-dimensional interaction analysis of FoHOS3 (A) and FoHOS2 (B) with histone acetylation modifications using LigPlot. **fig. S6**. LigPlot analysis of molecular docking interactions between eight histone acetylation modifications and FoRPD3. **fig. S7**. Two-dimensional visualization of docking complexes formed by FoSIR7 (A) and FoHST4 (B) with histone acetylation modifications. **fig. S8**. Molecular interaction profiles of histone acetylation modifications with FoHST3 (A) and FoSIR2 (B) through LigPlot analysis. **fig. S9**. Docking interface analysis of eight histone acetylation modifications with FoHST2 (A) and FoSIR5 (B). **fig. S10**. Comprehensive LigPlot mapping of FoSIR4 interactions with histone acetylation modifications. **table SS1**. Primer sequences for RT-qPCR amplification of FoHDAC genes. **table S2**. Conserved protein domains associated with HDAC function and classification in *F. oxysporum*. **table S3**. Architectural features of the conserved motifs in the FoHDAC gene family. **table S4**. Enriched GO (Gene Ontology) terms and FoHDAC genes in *F. oxysporum*. https://binary.pensoft.net/file/1522569This dataset is made available under the Open Database License (http://opendatacommons.org/licenses/odbl/1.0/). The Open Database License (ODbL) is a license agreement intended to allow users to freely share, modify, and use this Dataset while maintaining this same freedom for others, provided that the original source and author(s) are credited. Hong-Xin Liao, Jin-Rui Wen, Hong-Mei Shi, Huan-Qi Cun, Yun-Ju Hong, Zhang-Feng Hu, Fu-Rong Xu, Sulukkana Noiprasert, Kanyaphat Apiwongsrichai, Xiao-Yun Liu, Xian Dong
