# Supplementary material for: Genome-wide identification and expression profiling of the histone deacetylase gene family in Fusarium oxysporum
Source: IMA Fungus. 2026 Jan 29;17:e168980. doi: 10.3897/imafungus.17.168980 (PMC12877776; doi:10.3897/imafungus.17.168980)
Supplement: Supplementary material 1 — Additional information [file imafungus-17-e168980-s001.docx]

**Genome-Wide Identification and Expression Profiling of the Histone Deacetylase Gene Family in *Fusarium oxysporum***

Hong-Xin Liao^1, 2^, Jin-Rui Wen^1, 3^, Hong-Mei Shi^1^, Huan-Qi Cun^1^, Yun-Ju Hong^1^, Zhang-Feng Hu^2^, Fu-Rong Xu^1^, Sulukkana Noiprasert^4^, Kanyaphat Apiwongsrichai^4^, Xiao-Yun Liu^2*^, Xian Dong^1*^

1. Yunnan Key Laboratory of Chinese Medicine Processing, Yunnan University of Chinese Medicine, Kunming, China;
2. College of Life Sciences, Hubei Engineering Research Center for Protection and Utilization of Special Biological Resources in the Hanjiang River Basin/Jianghan University, Wuhan 430056, People’s Republic of China;
3. Key Laboratory of Soybean Disease and Pest Control (Ministry of Agriculture and Rural Affairs), Nanjing Agricultural University, Nanjing 210095 Jiangsu, China;
4. School of Integrative Medicine, Mae Fah Luang University, 333 Moo 1, Thasud, Muang, Chiang Rai 57100, Thailand.

* Corresponding author: [dongxian_1655129@163.com;](mailto:dongxian_1655129@163.com;) [liuxiaoyun@jhun.edu.cn](mailto:liuxiaoyun@jhun.edu.cn).

**Abstract:** Histone deacetylases (HDACs) are key epigenetic regulators governing chromatin structure and gene expression, playing critical roles in growth, development, virulence, and multi-stress resistance of plant-pathogenic fungi. Despite their importance, the HDAC gene family (FoHDACs) in *Fusarium oxysporum* remains poorly characterized. Through genome-wide analysis, we identified 11 *FoHDAC* genes, phylogenetically classified into three subfamilies: Class I (2 genes), Class II (2 genes), and SIR2 (7 genes). Subcellular localization predicted 6 in the nucleus, 3 in the cytoplasm, and 2 in mitochondria, indicating functional diversity across organelles. Structural analyses revealed conserved domains/motifs specific to each subfamily. Genes showed asymmetric distribution across 6 chromosomes with no recent duplication events. Promoter analysis identified 22 putative *cis*-elements, including antioxidant (ARE, as-1) and stress response elements (STRE), linking FoHDACs to development and environmental responses. Functional annotation highlighted putative roles in transcriptional regulation, macromolecular catabolism, and heterochromatin assembly beyond core HDAC activity. Molecular docking showed binding affinities < -5 kcal/mol with significant differences across subfamilies. RT-qPCR revealed stage-specific expression: 8 genes peaked in dormant conidia, were suppressed during germination, and recovered during growth/sporulation; 2 showed continuous activation, and 1 was sporulation-specific. Abiotic stresses induced stimulus-dependent regulation, e.g., 33.67-fold repression of *FoHST3* under salt stress and >100-fold induction of *FoHOS3* under cold stress. Collectively, our findings reveal that FoHDACs exhibit substantial functional diversity, forming a sophisticated regulatory network mediating fungal development and environmental adaptation.

**Keywords:** Histone deacetylase; *Fusarium oxysporum*; Genome-wide identification; Environmental stress response; Epigenetic regulation.

**
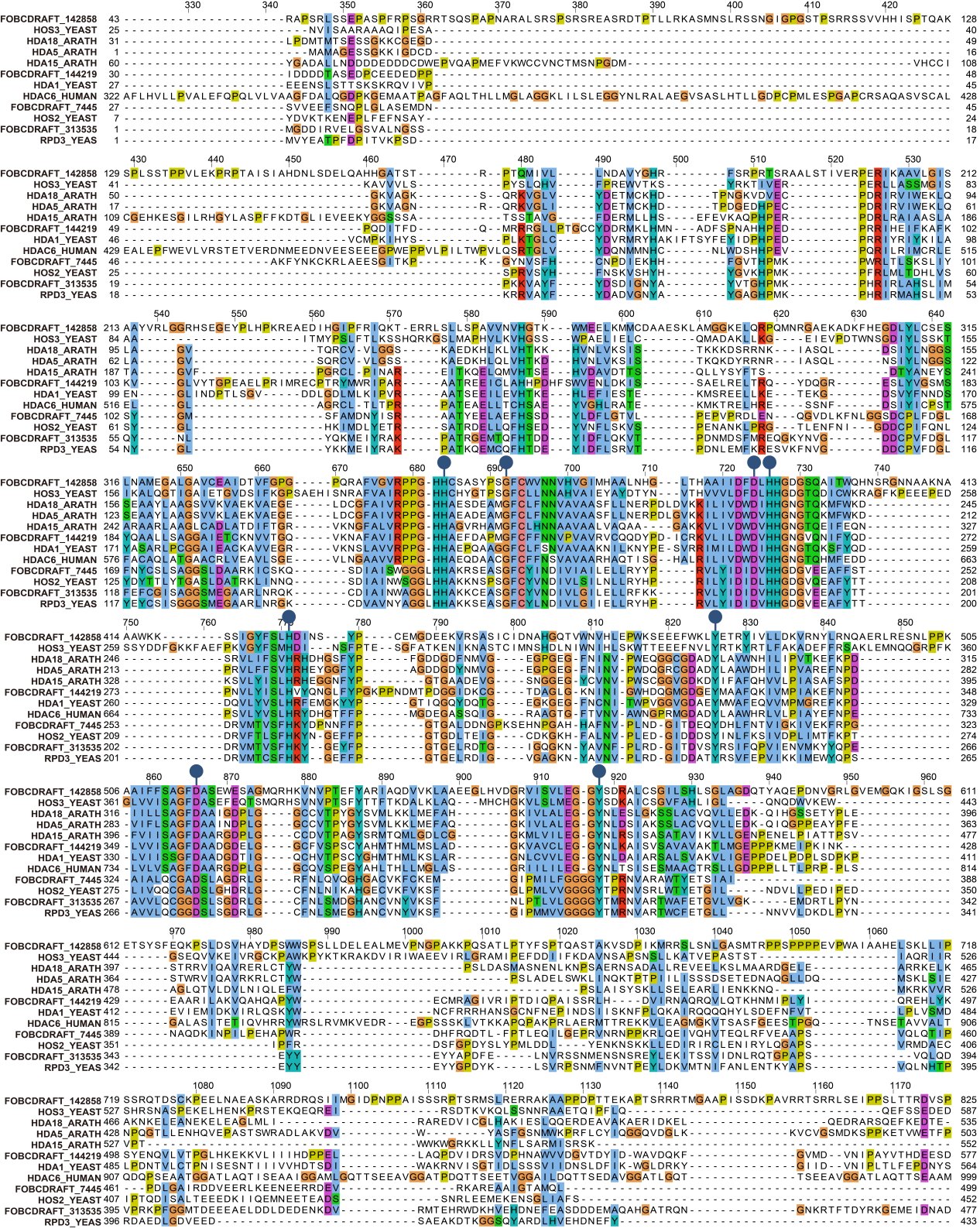
Figure S1.** Sequence alignment of HDAC class subfamily members. Multiple protein sequence alignment was performed using Jalview software (version 2.11.1.4). Conserved residues are indicated with blue lollipop symbols.


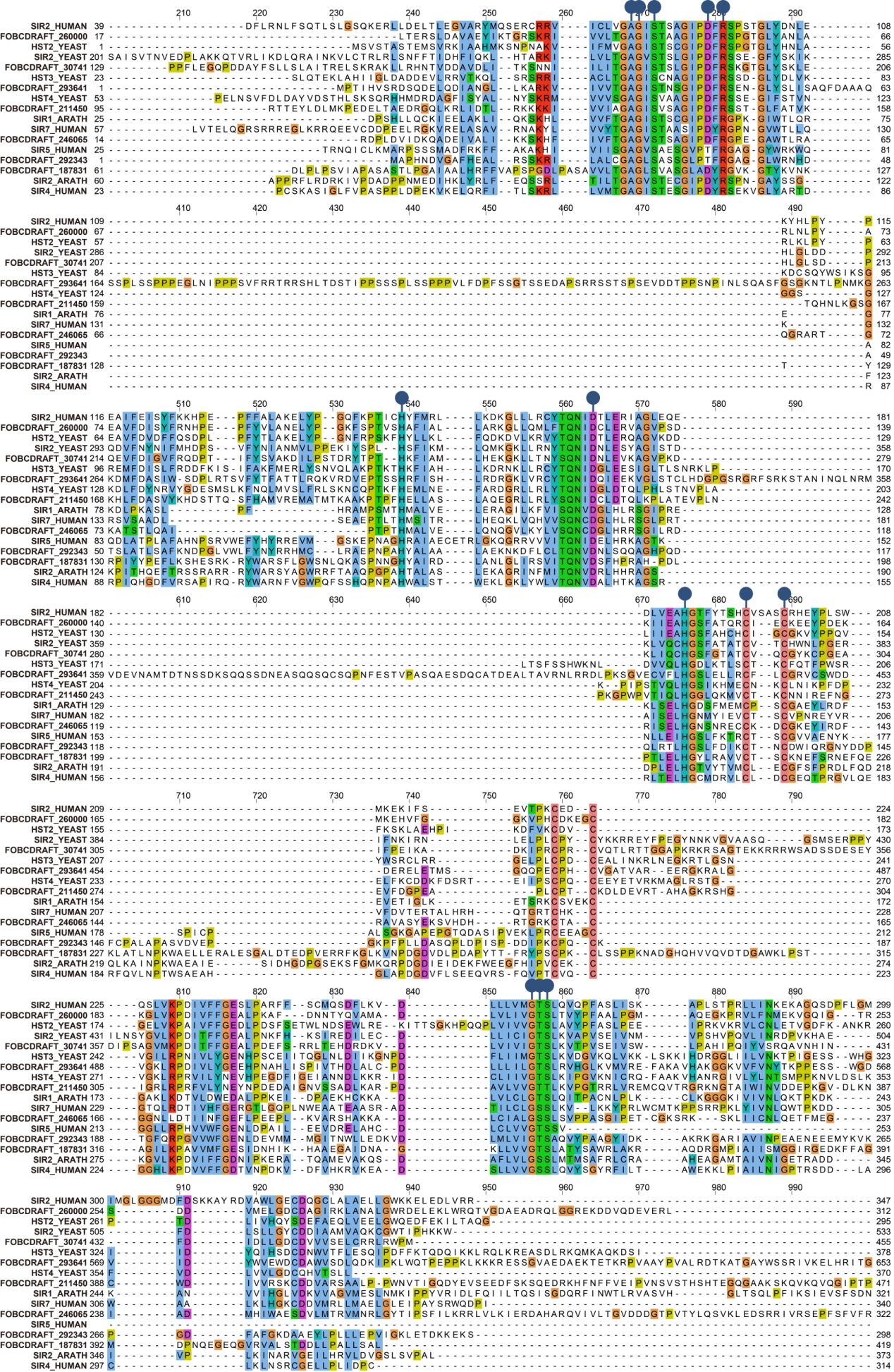


**Figure S2.** Sequence alignment of SIR2 class subfamily members. Multiple protein sequence alignment was performed using Jalview (version 2.11.1.4). Conserved residues are marked with blue lollipop symbols.

**
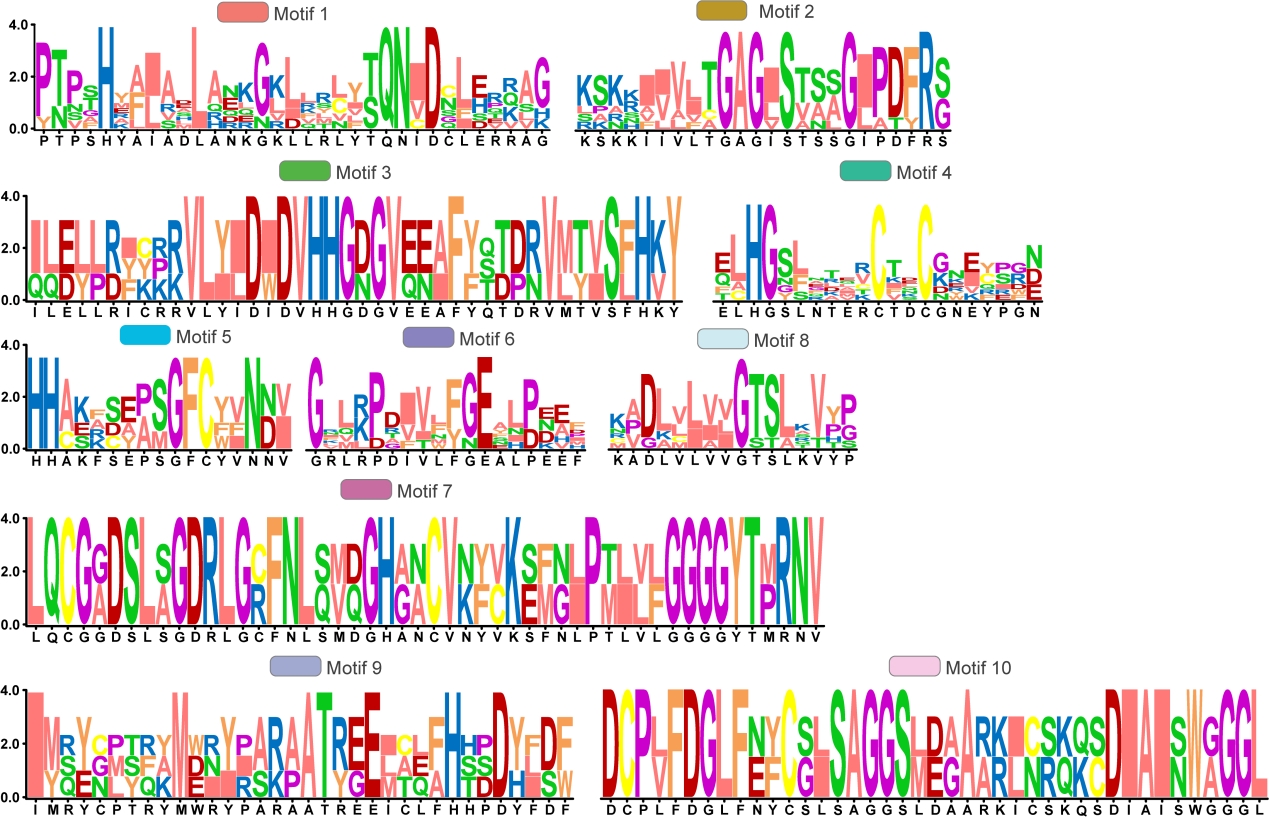
Figure S3.** FoHDAC amino acid frequency of 10 conserved motifs. Each letter represents an amino acid; the larger the letter in a motif, the higher is amino acid frequency.

**
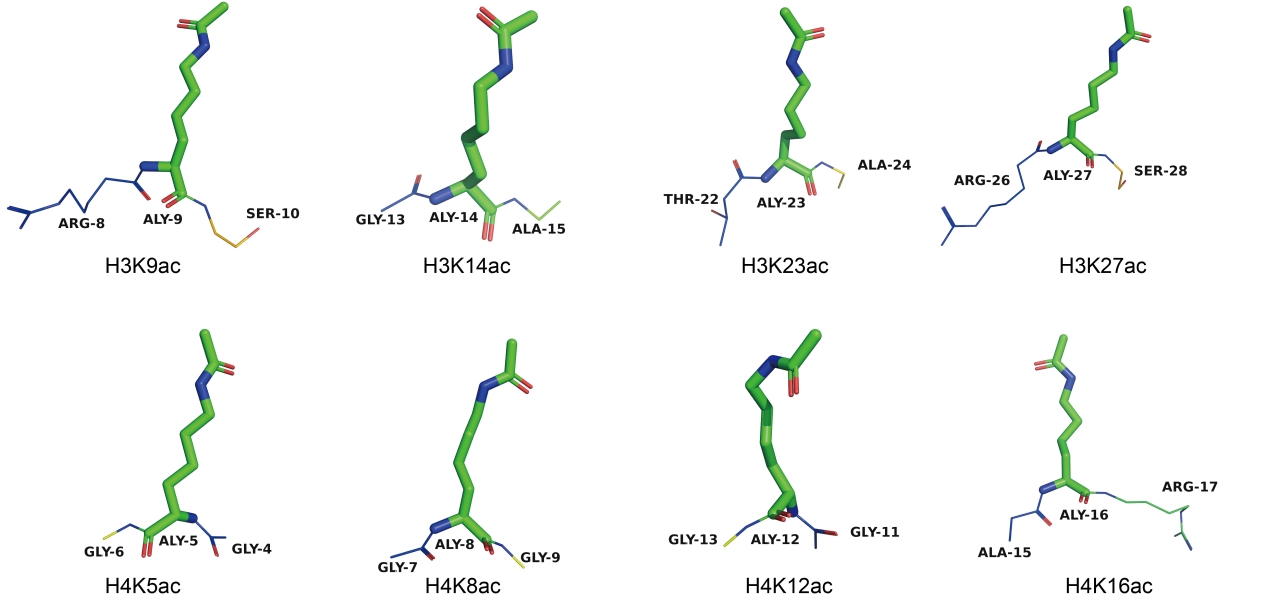
Figure S4.** Three-dimensional structural visualization of histone acetylation modifications in the FoHDAC catalytic domain using PyMOL v2.5. Acetylated lysine residues: green sticks; neighboring amino acids: green and blue lines.


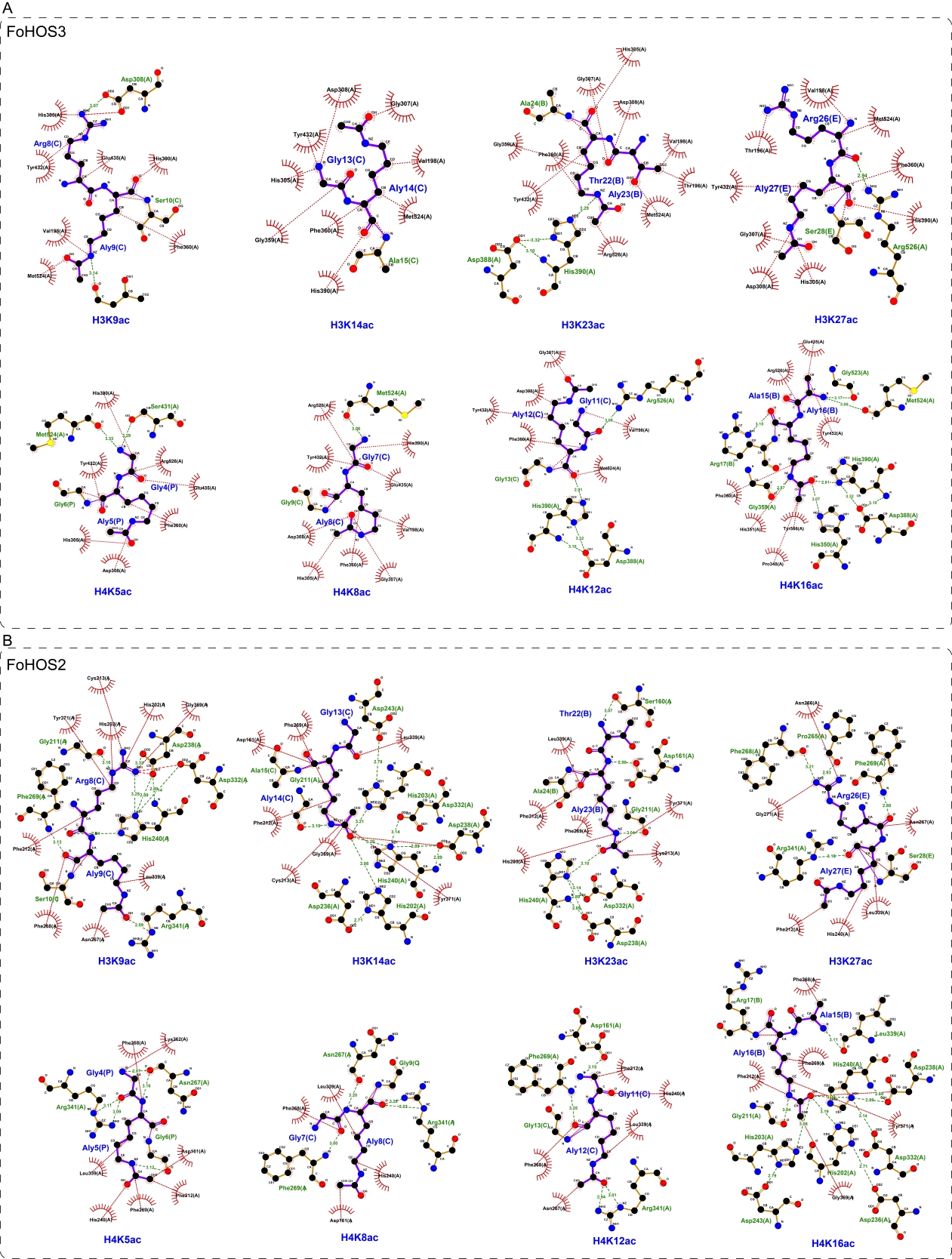
**Figure S5.** Two-dimensional interaction analysis of FoHOS3 (A) and FoHOS2 (B) with histone acetylation modifications using LigPlot. Hydrogen bonds are represented by dark lime green dashed lines, hydrophobic interactions by red arcs, catalytic residues by dark lime green, and acetylated sites by magenta.


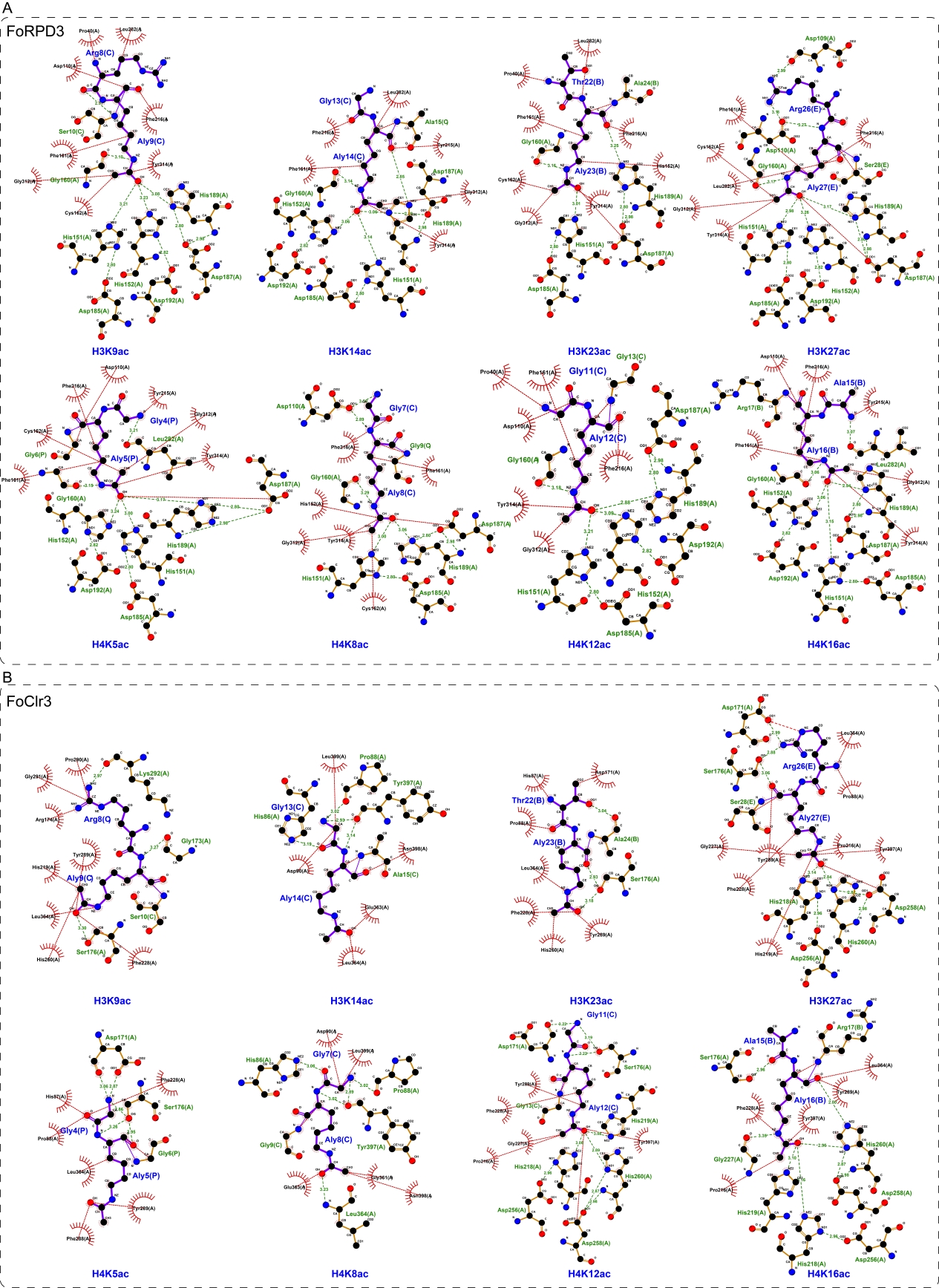
**Figure S6.** LigPlot analysis of molecular docking interactions between eight histone acetylation modifications and FoRPD3 (A)/FoClr3 (B). Key features: catalytic residues (dark lime green), acetylated sites (magenta), hydrogen bonds (dark lime green dashed lines), and hydrophobic interactions (red arcs).


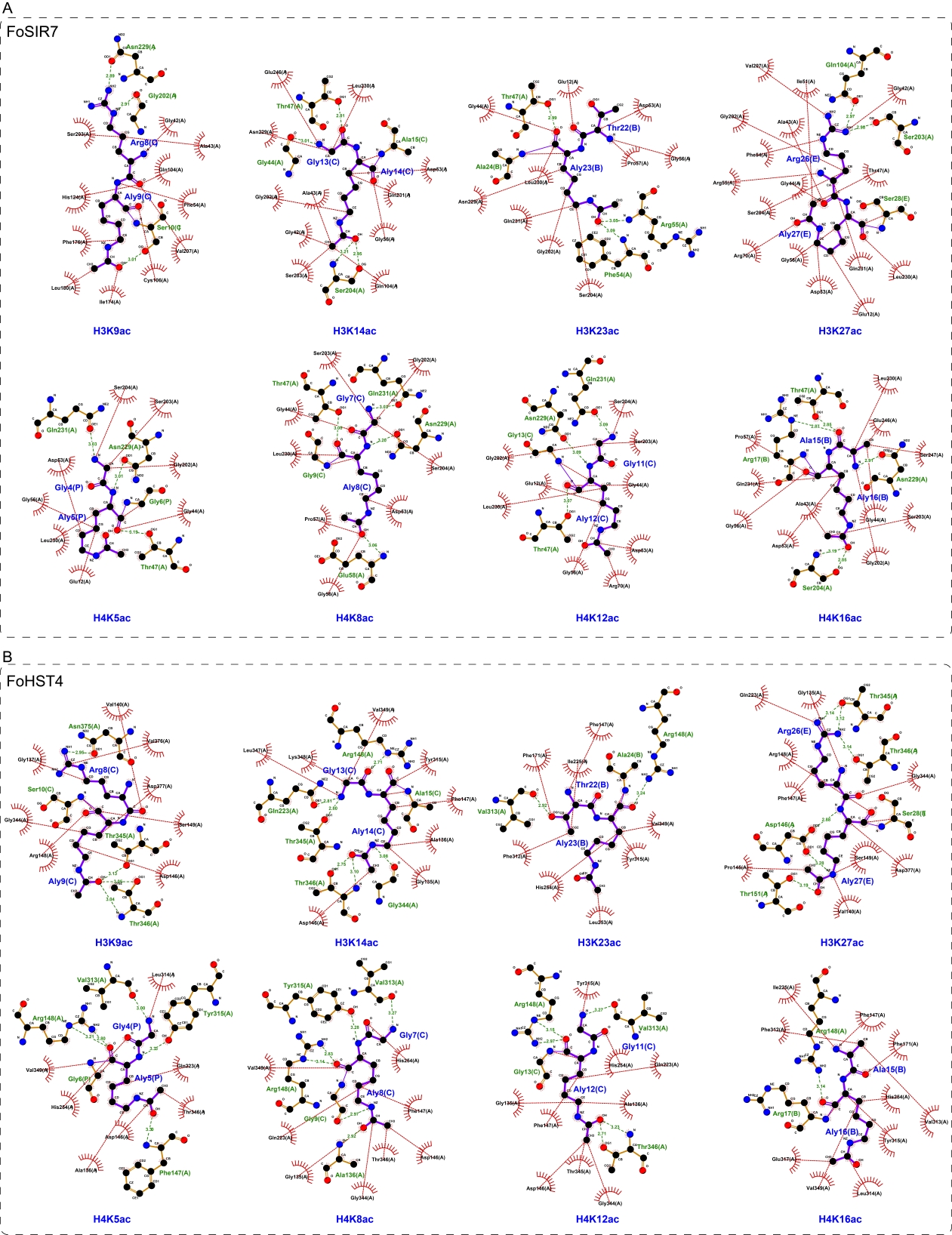
**Figure S7.** Two-dimensional visualization of docking complexes formed by FoSIR7 (A) and FoHST4 (B) with histone acetylation modifications. Diagrammatic representations: magenta = acetylated sites; dark lime green = catalytic residues; hydrogen bonds = dark lime green dashes; hydrophobic contacts = red arcs.


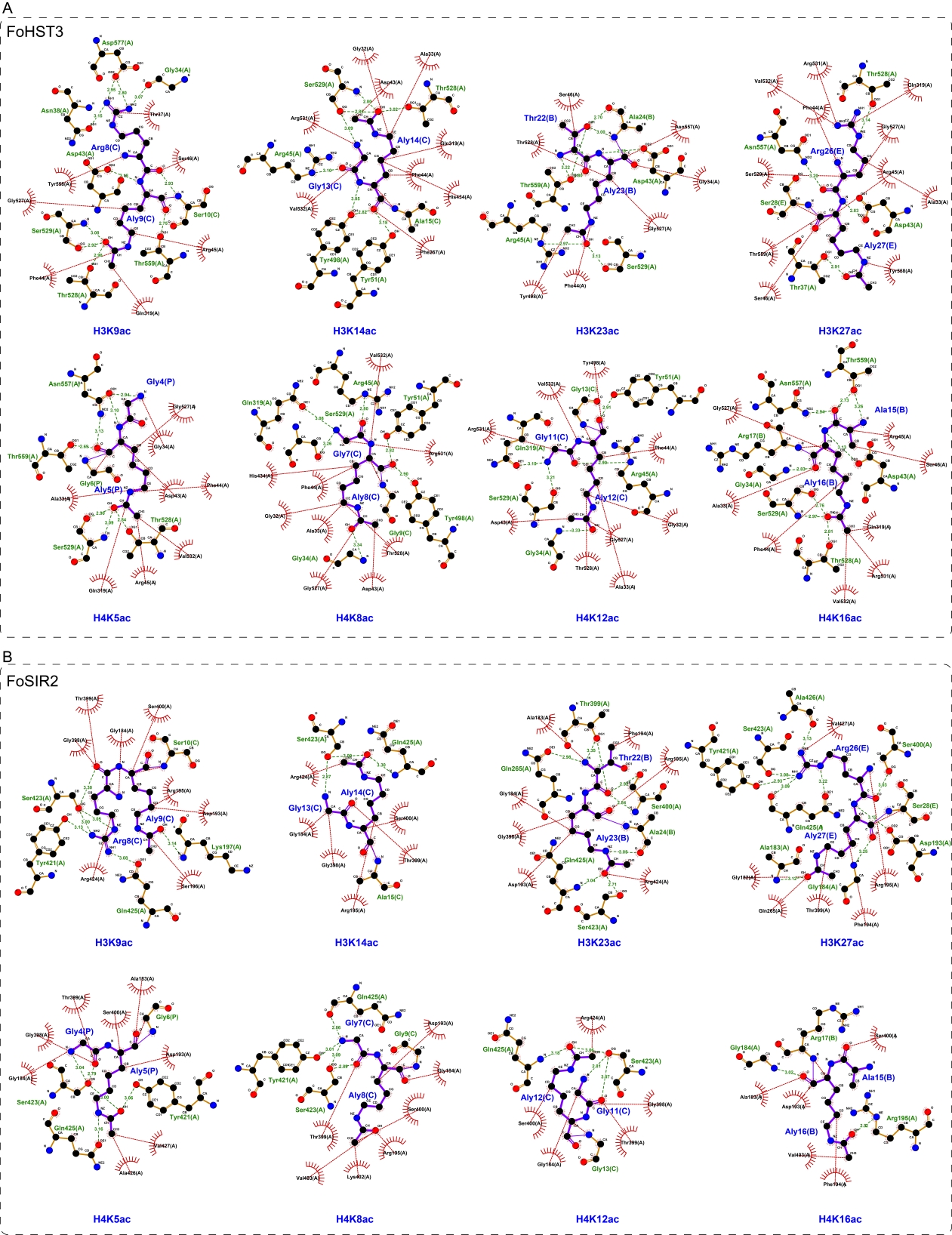
**Figure S8**. Molecular interaction profiles of histone acetylation modifications with FoHST3 (A) and FoSIR2 (B) through LigPlot analysis. Visualization scheme: catalytic residues (dark lime green), modification sites (magenta), hydrogen-bond networks (dark lime green dashed lines), and hydrophobic interfaces (red arcs).


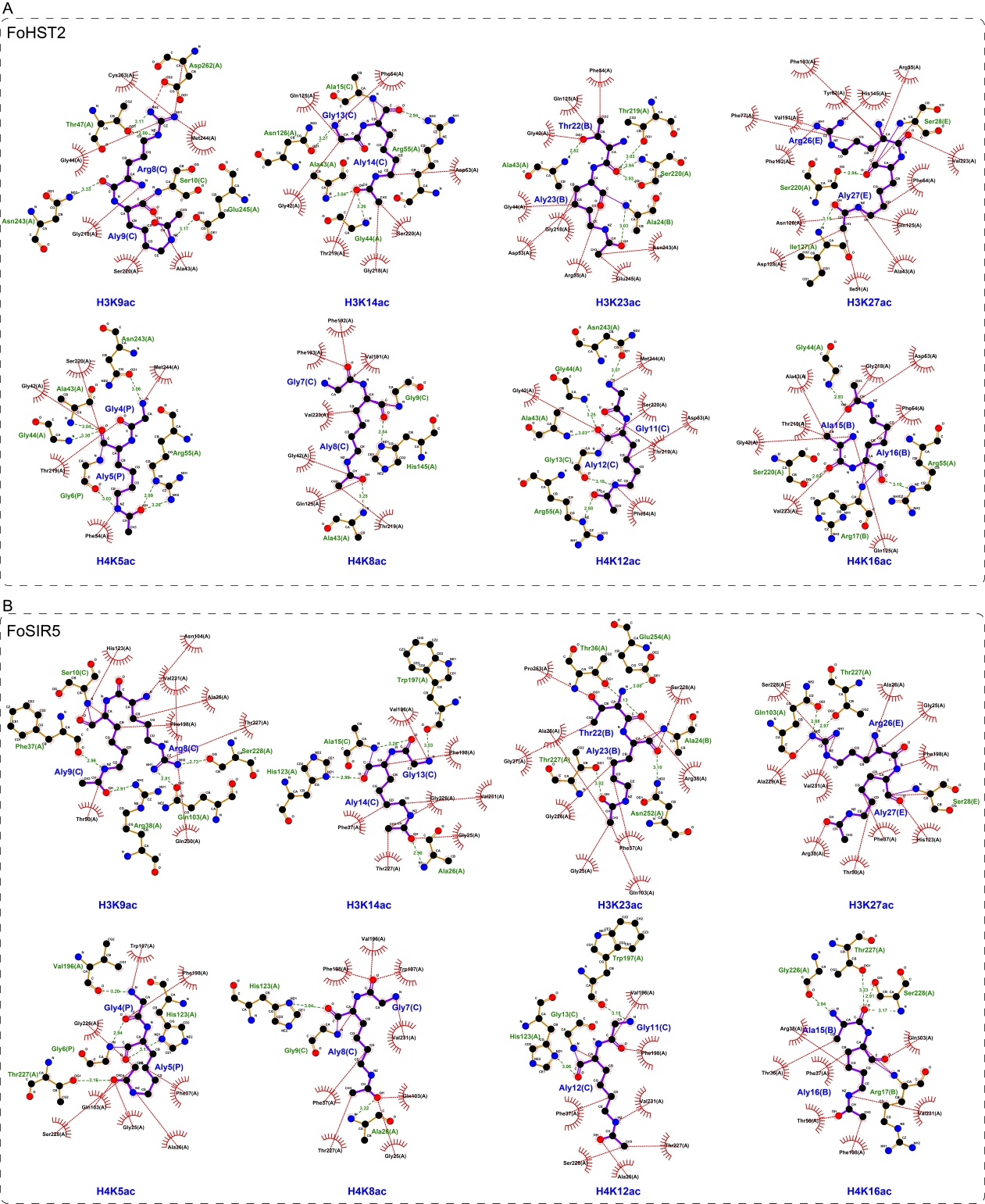
**Figure S9.** Docking interface analysis of eight histone acetylation modifications with FoHST2 (A) and FoSIR5 (B). Critical elements shown: acetyl-lysine positions (magenta), catalytic centers (dark lime green), hydrogen bonds (dark lime green dashes), and hydrophobic interaction zones (red arcs).


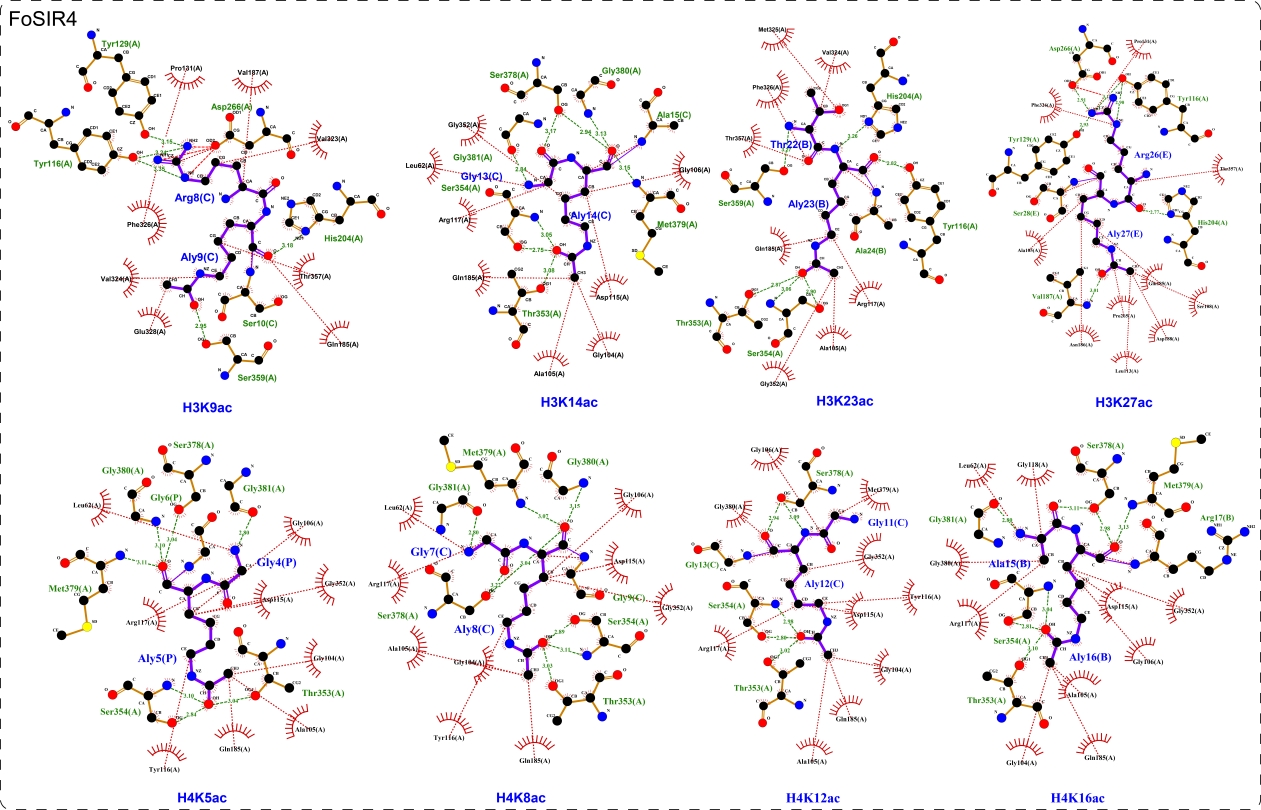
**Figure S10.** Comprehensive LigPlot mapping of FoSIR4 interactions with histone acetylation modifications. Annotated features: catalytic residues (dark lime green), acetylation sites (magenta), hydrogen bonds (dark lime green dashed lines), and hydrophobic contacts (red arcs).

| Table S1 Primer sequences for RT-qPCR amplification of FoHDAC genes | | |
| --- | --- | --- |
| Genes | Forward primer | Reverse primer |
| FoHOS3 | ACACAGGCATCAACGGCTAA | AGGCTTGCAGCTATCGGTTT |
| FoHOS2 | GATGCTTCTCAATGTCCGGATTG | GATAACTCGGTCGTTGAGGAGTTC |
| FoRPD3 | GTCATCCCATGAAGCCTCATCG | CGCATGAAGCTATCCATGTTGTC |
| FoClr3 | CATCCAATGAAGGATGCAGAAGC | CAACAGCCTGTTGGCAACAAAC |
| FoSIR7 | AGAAGGTGCTTGGACACTGAG | CAGGATGCCACTCCTTCGATG |
| FoHST4 | TAACCTGTTTCCCGTTGACGG | TATTATCGGCCAAGAGAAGGCC |
| FoHST3 | GGACGAACTACAAGACATCGCC | GAATTGAGCGCTGATTAGGGAG |
| FoSIR2 | CACTCGCTGAACTTGAGGAGAG | GAAGTCTGCTAGCTTCTTCAGGTG |
| FoHST2 | GAGTATATCAAGACGGGTCGGAG | GCAAGGTTGGCATAAAGACCTG |
| FoSIR5 | ATGGCTCCTCATAACGACGTAG | TCATGATTTCGCCAAAGACCG |
| FoSIR4 | ATGCCACATGAACCCCTACTTG | GTCCGTATAAGGGATCCTGAGCA |

| Table S2 Conserved protein domains associated with HDAC function and classification in *F. oxysporum.* | | | | |
| --- | --- | --- | --- | --- |
| Family Name | Functional description | Locus_tag | Position in Protein | |
|  |  |  | From | to |
| PHA03307 superfamily | transcriptional regulator ICP4 | FOBCDRAFT_142858 | 2 | 136 |
| PHA03247 superfamily | large tegument protein UL36 | FOBCDRAFT_142858 | 751 | 1068 |
| LGT superfamily | prolipoprotein diacylglyceryl transferase | FOBCDRAFT_313535 | 477 | 640 |
|  |  | FOBCDRAFT_260000 | 305 | 436 |
| Arb2 superfamily | necessary for centromeric heterochromatin silencing | FOBCDRAFT_144219 | 467 | 721 |

| Table S3 Architectural features of the conserved motifs in the FoHDAC gene family | | | | | |
| --- | --- | --- | --- | --- | --- |
|  | Sequences | Size | Domain | Conserved Residues | Function |
| motif1 | PTPSHYFJAELAEKGKLLRLYTQNIDNLERKAG | 33 | [DHS-like NAD/FAD-binding](https://www.ebi.ac.uk/QuickGO/GTerm?id=GO:0070403) | NP_BIND: NAD | [NAD+ binding](https://www.ebi.ac.uk/QuickGO/GTerm?id=GO:0070403) |
| motif2 | KSKKIIVJTGAGISTSAGIPDFRS | 24 | [DHS-like NAD/FAD-binding](https://www.ebi.ac.uk/QuickGO/GTerm?id=GO:0070403) | NP_BIND: NAD | unknow |
| motif3 | ILELLRFYKRVLYIDIDVHHGDGVEEAFYSTDRVMTVSFHKY | 42 | histone deacetylase | METAL: Divalent metal cation | histone deacetylase activity |
| motif4 | ZLHGSLETVVCTECGNEYPGN | 21 | unknow | unknow | unknow |
| motif5 | HHAKKSEPSGFCYVNDI | 17 | histone deacetylase | ACT_SITE: Proton acceptor | unknow |
| motif6 | GILKPDIVLFGEALPEEF | 18 | DHS-like NAD/FAD-binding domain | unknow | unknow |
| motif7 | LQCGGDSLAGDRLGCFNLQMDGHGNCVKFCKEFGJPMJLFGGGGYTPRNV | 50 | histone deacetylase | METAL: Divalent metal cation | unknow |
| motif8 | KADLVLVVGTSLKVYP | 16 | unknow | NP_BIND: NAD | unknow |
| motif9 | IMRYGLSRKMDRYPARAATREEJAQFHSPDYLDF | 34 | histone deacetylase | unknow | unknow |
| motif10 | DCPLFDGLFEFCGJSAGGSLEGARKJCRKKCDIAINWGGGL | 41 | histone deacetylase | unknow | unknow |

| Table S4 Enriched GO (Gene Ontology) terms and FoHDAC genes in *F. oxysporum* | | | | |
| --- | --- | --- | --- | --- |
|  | Term ID | Term description | p-value | Gene name |
| Molecular Function | GO:0033558 | Protein lysine deacetylase activity | 3.10e-30 | FoHST4, FoHOS3, FoHST3, FoClr3, FoHOS2, FoRPD3, FoSIR2, FoSIR7, FoSIR4, FoSIR5, FoHST2 |
|  | GO:0004407 | Histone deacetylase activity | 1.10e-23 | FoHST4, FoHOS3, FoHST3, FoClr3, FoHOS2, FoRPD3, FoSIR2, FoSIR7, FoHST2 |
|  | GO:0016811 | Hydrolase activity, acting on carbon-nitrogen (but not peptide) bonds, in linear amides | 3.53e-20 | FoHST4, FoHOS3, FoHST3, FoClr3, FoHOS2, FoRPD3, FoSIR2, FoSIR7, FoSIR5, FoHST2 |
|  | GO:0070403 | NAD+ binding | 5.64e-16 | FoHST4, FoHST3, FoSIR2, FoSIR7, FoSIR4, FoSIR5, FoHST2 |
|  | GO:0034979 | NAD-dependent protein deacetylase activity | 4.49e-15 | FoHST4, FoHST3, FoSIR2, FoSIR7, FoSIR4, FoHST2 |
|  | GO:0017136 | NAD-dependent histone deacetylase activity | 3.13e-12 | FoHST4, FoHST3, FoSIR2, FoSIR7, FoHST2 |
|  | GO:0046969 | NAD-dependent histone deacetylase activity (H3-K9 specific) | 0.0003 | FoSIR2, FoSIR7 |
|  | GO:0016740 | Transferase activity | 0.0019 | FoHST4, FoHST3, FoSIR2, FoSIR7, FoSIR4, FoSIR5, FoHST2 |
| Biological Process | GO:0035601 | Protein deacylation | 3.50e-17 | FoHST4, FoHST3, FoClr3, FoHOS2, FoRPD3, FoSIR7, FoSIR4, FoSIR5 |
|  | GO:0006476 | Protein deacetylation | 2.35e-12 | FoHST4, FoHST3, FoClr3, FoRPD3, FoSIR7, FoSIR4 |
|  | GO:0016575 | Histone deacetylation | 9.91e-10 | FoHST4, FoHST3, FoClr3, FoRPD3, FoSIR7 |
|  | GO:0006325 | Chromatin organization | 3.50e-08 | FoHST4, FoHST3, FoClr3, FoHOS2, FoRPD3, FoSIR2, FoHST2 |
|  | GO:0010605 | Negative regulation of macromolecule metabolic process | 2.52e-07 | FoHST4, FoHST3, FoClr3, FoRPD3, FoSIR2, FoSIR7, FoHST2 |
|  | GO:0031507 | Heterochromatin assembly | 2.52e-07 | FoHST4, FoHST3, FoClr3, FoSIR2, FoHST2 |
|  | GO:0006357 | Regulation of transcription by RNA polymerase II | 7.10e-07 | FoHST4, FoHOS3, FoHST3, FoClr3, FoHOS2, FoRPD3, FoSIR2, FoSIR7 |
|  | GO:0010468 | Regulation of gene expression | 1.97e-06 | FoHST4, FoHOS3, FoHST3, FoClr3, FoHOS2, FoRPD3, FoSIR2, FoSIR7, FoHST2 |
|  | GO:0006996 | Organelle organization | 4.91e-06 | FoHST4, FoHST3, FoClr3, FoHOS2, FoRPD3, FoSIR2, FoSIR7, FoHST2 |
|  | GO:0022607 | Cellular component assembly | 4.37e-05 | FoHST4, FoHST3, FoClr3, FoSIR2, FoSIR7, FoHST2 |
| Cellular Component | GO:0000785 | Chromatin | 3.62e-07 | FoHST4, FoHST3, FoClr3, FoRPD3, FoSIR2, FoSIR7 |
|  | GO:0031934 | Mating-type region heterochromatin | 3.62e-07 | FoHST4, FoHST3, FoClr3, FoSIR2 |
|  | GO:0000118 | Histone deacetylase complex | 6.04e-05 | FoClr3, FoHOS2, FoRPD3 |
|  | GO:0033553 | rDNA heterochromatin | 0.00074 | FoClr3, FoSIR2 |
|  | GO:0005634 | Nucleus | 0.0019 | FoHST4, FoHOS3, FoHST3, FoClr3, FoHOS2, FoRPD3, FoSIR2, FoSIR7, FoHST2 |
|  | GO:0043231 | Intracellular membrane-bounded organelle | 0.002 | FoHST4, FoHOS3, FoHST3, FoClr3, FoHOS2, FoRPD3, FoSIR2, FoSIR7, FoSIR4, FoSIR5, FoHST2 |
|  | GO:0099115 | Chromosome, subtelomeric region | 0.0074 | FoClr3, FoSIR2 |
|  | GO:0005721 | Pericentric heterochromatin | 0.011 | FoClr3, FoSIR2 |
